# Nighttime home blood pressure lowering effect of esaxerenone in patients with uncontrolled nocturnal hypertension: the EARLY-NH study

**DOI:** 10.1038/s41440-023-01292-0

**Published:** 2023-05-12

**Authors:** Kazuomi Kario, Masafumi Nishizawa, Mitsutoshi Kato, Hajime Ishii, Kazuaki Uchiyama, Michiaki Nagai, Nobuo Takahashi, Taro Asakura, Toshihiko Shiraiwa, Tetsuro Yoshida, Mizuki Kaneshiro, Takashi Taguchi, Kazuhito Shiosakai, Kotaro Sugimoto

**Affiliations:** 1 Division of Cardiovascular Medicine, Department of Medicine, Jichi Medical University School of Medicine, Shimotsuke, Japan; 2 Minamisanriku Hospital, Motoyoshi, Japan; 3 Kato Clinic of Internal Medicine, Tokyo, Japan; 4 Kashinoki Internal Medicine, Date, Japan; 5 Uchiyama Clinic, Joetsu, Japan; 6 Department of Cardiology, Hiroshima City Asa Hospital, Hiroshima, Japan; 7 Takahashi Family Clinic, Nagoya, Japan; 8 Tsuruma Kaneshiro Diabetes Clinic, Yamato, Japan; 9 Shiraiwa Medical Clinic, Kashiwara, Japan; 10 Department of Cardiovascular Medicine, Onga Nakama Medical Association Onga Hospital, Onga, Japan; 11 Primary Medical Science Department, Daiichi Sankyo Co., Ltd., Tokyo, Japan; 12 Data Intelligence Department, Daiichi Sankyo Co., Ltd., Tokyo, Japan

**Keywords:** Esaxerenone, Home blood pressure, Mineralocorticoid receptor blocker, Nocturnal hypertension, Uncontrolled hypertension

## Abstract

There is limited evidence on the blood pressure (BP)-lowering effect of esaxerenone on home BP, including nighttime BP. Using two newly developed nocturnal home BP monitoring devices (brachial and wrist), this multicenter, open-label, prospective study investigated the nighttime home BP-lowering effect of esaxerenone in patients with uncontrolled nocturnal hypertension being treated with an angiotensin receptor blocker (ARB) or calcium-channel blocker (CCB). In total, 101 patients were enrolled. During the 12-week study period, change in nighttime home systolic/diastolic BP from baseline to end of treatment measured by the brachial device was −12.9/−5.4 mmHg in the total population and −16.2/−6.6 and −10.0/−4.4 mmHg in the ARB and CCB subcohorts, respectively (all *p* < 0.001). For the wrist device, the change was −11.7/−5.4 mmHg in the total population and −14.6/−6.2 and −8.3/−4.5 mmHg in each subcohort, respectively (all *p* < 0.001). Similar significant reductions were shown for morning and bedtime home BP and office BP. Urinary albumin-to-creatinine ratio, N-terminal pro-brain natriuretic peptide, and cardio-ankle vascular index improved in the total population and each subcohort. Incidences of treatment-emergent adverse events (TEAEs) and drug-related TEAEs were 38.6% and 16.8%, respectively; most were mild or moderate. The most frequent drug-related TEAEs were associated with serum potassium elevation (hyperkalemia, 9.9%; blood potassium increased, 3.0%); however, no new safety concerns were raised. Esaxerenone was effective in lowering nighttime home BP as well as morning and bedtime home BP and office BP, safe, and showed organ-protective effects in patients with uncontrolled nocturnal hypertension. Caution is warranted regarding elevated serum potassium levels.

**Figure:**
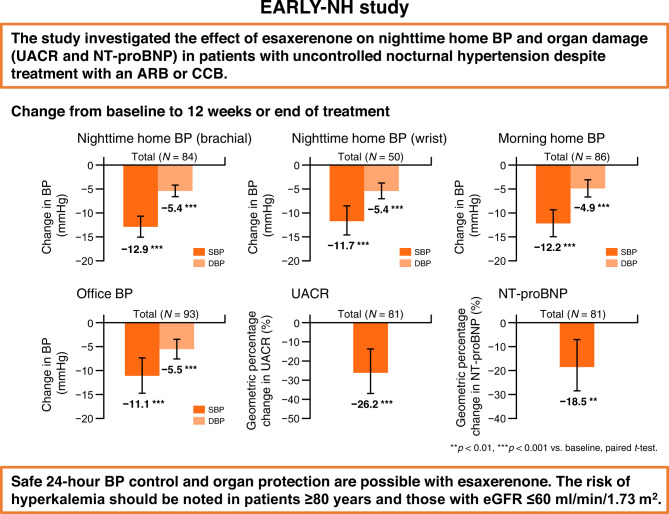
This study investigated the effect of esaxerenone on nighttime home BP and organ damage (UACR and NT-proBNP) in patients with uncontrolled nocturnal hypertension despite treatment with an ARB or CCB. Our results show that safe 24-h BP control and organ protection are possible with esaxerenone.

This study investigated the effect of esaxerenone on nighttime home BP and organ damage (UACR and NT-proBNP) in patients with uncontrolled nocturnal hypertension despite treatment with an ARB or CCB. Our results show that safe 24-h BP control and organ protection are possible with esaxerenone.

## Abstract (web summary)

This study investigated the effect of esaxerenone on nighttime home BP and organ damage (UACR and NT-proBNP) in patients with uncontrolled nocturnal hypertension despite treatment with an ARB or CCB. Our results show that safe 24-h BP control and organ protection are possible with esaxerenone.

## Introduction

Nocturnal hypertension is mainly caused by increased circulating blood volume associated with cardiac or renal failure, advanced structural vascular disease, and excessive salt intake [1, 2]. More than 45% of hypertensive patients on hypertensive treatment remain with uncontrolled nocturnal hypertension [3]. Identifying patients with nocturnal hypertension is difficult because of the high patient burden of measuring nighttime blood pressure (BP) with ambulatory BP monitoring (ABPM). However, recent development of brachial and wrist type nocturnal home BP monitoring (HBPM) devices makes taking nighttime BP measurements easier [2, 4–7].

Among the various types of hypertension, adequate control of nocturnal hypertension is particularly important because it is associated with a high risk of organ damage and cardiovascular events [8–11]. Many patients with nocturnal hypertension take angiotensin receptor blockers (ARBs) or calcium-channel blockers (CCBs), the most frequently prescribed antihypertensive drugs among Japanese patients [12]. However, because of its etiology, many of these patients also have comorbidities such as chronic kidney disease and diabetes mellitus (DM), making control of nocturnal hypertension difficult with antihypertensive drug monotherapy. To reduce the risk of organ damage, a combination of antihypertensive medication other than ARB and CCB is required.

Mineralocorticoid receptor (MR) blockers (MRBs) are expected to exert antihypertensive and organ-protective effects in MR-associated hypertension caused by lifestyle factors such as excessive salt intake, DM, and obesity [13]. In a long-term study in patients with essential hypertension, esaxerenone, a next-generation MRB with high selectivity, administered alone or in combination was found to have antihypertensive effects on nighttime BP and normalization of dipping patterns, and to reduce N-terminal pro-brain natriuretic peptide (NT-proBNP) levels [14, 15]. In hypertensive patients with albuminuria and DM, consistent urinary albumin-to-creatinine ratio (UACR)-lowering effects have been observed in addition to the antihypertensive effects [16–19]. However, there is insufficient clinical evidence on the effect of esaxerenone in patients with nocturnal hypertension.

This study aimed to investigate the nighttime home BP-lowering effect of esaxerenone in patients with uncontrolled nocturnal hypertension being treated with an ARB or CCB, using two newly developed brachial and wrist nocturnal HBPM devices.

## Methods

### Study design and treatment

This was a multicenter, open-label, prospective interventional study (Supplementary Fig. 1) conducted from April 2021 to March 2022 at 17 study centers in Japan (Supplementary Table 1). This study had a 4-week observation period and a 12-week treatment period. Esaxerenone was administered orally once daily according to the package insert [20]. The starting dose was 2.5 mg, which could be titrated to 5 mg if the response was inadequate. In patients with moderate renal impairment (creatinine-based estimated glomerular filtration rate [eGFRcreat] 30 to <60 ml/min/1.73 m^2^) or DM with albuminuria or proteinuria, esaxerenone was started at 1.25 mg, and the dose was increased to 2.5 mg from week 4 onwards depending on the patient’s condition (e.g., serum potassium level). If the response was inadequate, the dose could be increased to 5 mg at week 8.

Serum potassium levels were measured at 2 and 4 weeks after dose adjustments, according to the esaxerenone package insert [20]. If the serum potassium level was >5.0 mEq/l at each visit, dose reduction was considered; at ≥5.5 mEq/l, the dose was reduced or treatment was discontinued; at ≥6.0 mEq/l, treatment was discontinued immediately. Basal antihypertensive drugs (ARB or CCB) were continued without any changes in dose during the study period.

The protocol was approved by the Certified Review Board of Hattori Clinic (CRB3180027) and prospectively registered with the Japan Registry of Clinical Trials (jRCTs031200364; https://jrct.niph.go.jp/en-latest-detail/jRCTs031200364). The study was conducted in accordance with the Declaration of Helsinki and the Clinical Trials Act in Japan. All patients provided written informed consent.

### Patients

The inclusion criteria were as follows: patients ≥20 years at the time of informed consent, who were administered a constant dose of one ARB or one CCB for 4 weeks prior to the start of esaxerenone, and with a nighttime systolic BP (SBP) measured with a brachial device of ≥120 mmHg (5-day average during the observation period). This reference value for SBP was set according to the definition of nocturnal hypertension in the Japanese Society of Hypertension 2019 guideline (SBP of ≥120 mmHg and/or diastolic BP [DBP] of ≥70 mmHg) [21].

The major exclusion criteria were as follows: diagnosis of secondary hypertension (e.g., endocrine hypertension, pregnancy-induced hypertension, and hypertension due to having a solitary kidney), or malignant hypertension; hyperkalemia or serum potassium levels >5.0 mEq/l at the end of the observation period; myocardial infarction, angina pectoris, chronic atrial fibrillation, cerebral infarction, cerebral hemorrhage, subarachnoid hemorrhage, or transient ischemic attack within 12 weeks prior to obtaining consent; use of prohibited concomitant medications within 4 weeks prior to the start of the observation period; severe renal impairment (eGFRcreat <30 ml/min/1.73 m^2^ at the end of the observation period); severe hepatic dysfunction; or patients working a night shift at least 3 days a week in a shift-work system.

### BP measurements

Home BP was automatically measured using brachial (OMRON HEM-9700T) or wrist (OMRON HEM-9601T) HBPM devices; all data were collected electronically. Nighttime BP was measured at 02:00, 03:00, and 04:00 h with both the brachial and wrist devices (brachial, from week −1 to 0 and weeks 11–12; wrist, from week −2 to −1 and weeks 10–11) (Supplementary Fig. 1). Measurements were continued in the supine position even after awakening at night. Morning home BP was measured with the brachial device after urination within 1 h after waking up and before breakfast, medication, and caffeine intake. Bedtime home BP was measured with the brachial device >1 h after bathing, drinking, or caffeine intake before bedtime. Office BP was measured twice at each visit (at baseline; 4, 8, and 12 weeks; and at discontinuation), and the average of the two measurements was used. Office BP was measured after at least 5 min of rest in a sitting position.

### Measurement of other outcomes

Albumin and creatinine concentrations from spot urine samples were measured in a central laboratory (SRL, Inc., Tokyo, Japan) at baseline, 12 weeks, and at discontinuation. UACR was calculated as follows: UACR (mg/gCr) = urinary albumin (µg/ml)/urinary creatinine (mg/dl) × 100. eGFRcreat was calculated as follows: 194 × serum creatinine^−1.094^ × age^−0.287^ (× 0.739 for women). Serum potassium and creatinine levels were measured at baseline; 2, 4, 6, 8, 10, and 12 weeks; and at discontinuation (measurements at 4 and 8 weeks were used to determine if dose escalation was needed; measurements at 6 and 10 weeks were used to confirm the safety of patients receiving dose escalations at 4 and 8 weeks, respectively). Plasma aldosterone concentration (PAC) and plasma renin activity (PRA) were measured at baseline, 12 weeks, and at discontinuation. Serum NT-proBNP levels and urinary sodium and potassium levels were also measured at the same timepoints as PAC and PRA in the central laboratory. Only in facilities where a vascular screening device was available, cardio-ankle vascular index (CAVI) was measured (supine position) at baseline, 12 weeks, and discontinuation, ≥4 h after meals and after ≥10 min of rest.

### Outcomes

The primary endpoint was the change in nighttime home SBP and DBP measured with the brachial device from baseline to the end of treatment (EOT). Key secondary endpoints were as follows: change from baseline to EOT in morning home, bedtime home, and office SBP/DBP; change from baseline to EOT in nighttime home SBP/DBP measured with the wrist device; time course of changes in morning home, bedtime home, and office BP; achievement rate of target BP levels (SBP/DBP, office BP <130/80 mmHg, home BP [morning and bedtime] <125/75 mmHg, and nighttime home BP <120/70 mmHg) [21] at week 12; and percentage change from baseline in UACR, NT-proBNP, and CAVI at week 12. Safety endpoints were treatment-emergent adverse events (TEAEs), time course of changes and change from baseline in eGFRcreat and serum potassium level, and proportions of patients with serum potassium levels ≥5.5 mEq/l and ≥6.0 mEq/l.

### Statistical methods

This was an exploratory study, and the number of cases was determined based on practicality; the analysis was not adjusted for multiplicity. Based on previous studies [14, 22], we assumed a change in nighttime home BP (SBP/DBP) in response to esaxerenone of −10.0/−5.0 mmHg and an SD of 19/11 mmHg. Thus, the power of detection is at least 93% for SBP and 84% for DBP when the number of patients for analysis is 45 and the significance level is set at 5% (two-sided). Assuming that five patients were excluded from the analyses, the target number of study patients was set at 100 (*n* = 50 each for the ARB and CCB cohorts).

Analyses were conducted in the total population and stratified by baseline antihypertensive drugs (ARB and CCB subcohorts). No statistical comparisons were performed between subcohorts. Efficacy was evaluated using the full analysis set (FAS), defined as all patients who met the inclusion criteria, received at least one dose of esaxerenone, and had at least one efficacy endpoint evaluation. The per-protocol set (PPS) was defined as FAS patients who adhered to the package insert of esaxerenone. Summary statistics of measurements at each time point and changes from baseline were calculated. For the percentage change in UACR, serum NT-proBNP, and CAVI, the point estimates and 95% confidence intervals (CIs) were calculated. Point estimates and 95% CIs for the difference between baseline and EOT values were calculated and compared using the paired *t*-test. EOT values were calculated by taking the average of measurements at the last two visits in the treatment period. The missing BP values at the EOT were imputed by the last observation carried forward method. Missing serum and urinary biomarkers and safety endpoints were not imputed.

Safety was evaluated using the safety analysis set, defined as all patients who received at least one dose of esaxerenone, and were summarized using descriptive statistics. TEAEs were coded by System Organ Class and Preferred Term according to the Medical Dictionary for Regulatory Activities version 24.1 (Japanese translation). All statistical analyses were performed using SAS software version 9.4 (SAS Institute Inc., Cary, NC, USA).

## Results

### Patients

Of 206 patients who provided informed consent, 105 were excluded (not nocturnal hypertension, *n* = 88; others, *n* = 17). In total, 101 patients were included in the safety analysis set (ARB, *n* = 48; CCB, *n* = 53) and 93 were included in the FAS (ARB, *n* = 45; CCB, *n* = 48). The PPS included 89 patients (ARB, *n* = 44; CCB, *n* = 45). A total of 82 patients (ARB, *n* = 38; CCB, *n* = 44) completed the study.

The mean age in the total population was 67.6 years; 50.5% were male; mean nighttime home BP with brachial sphygmomanometer (SBP/DBP), 132.4/78.4 mmHg; morning home BP, 143.8/86.7 mmHg; bedtime home BP, 135.1/80.5 mmHg; office BP, 147.2/84.1 mmHg; mean serum potassium level, 4.0 mEq/l; mean eGFRcreat, 69.7 ml/min/1.73 m^2^; mean UACR, 110.4 mg/gCr; and mean NT-proBNP, 83.7 pg/ml (Table 1). Compared with the CCB subcohort, the ARB subcohort tended to have a higher complication rate of type 2 DM (51.1% vs. 20.8%), higher UACR (ratio of ≥30, 35.6% vs. 25.0%), lower eGFRcreat (ratio of 30 to <60, 42.2% vs. 22.9%), and a longer history of hypertension; SBP and DBP were similar in both subcohorts.

**Table 1:** Baseline patient characteristics (full analysis set)

	Total *N* = 93	ARB subcohort *n* = 45	CCB subcohort *n* = 48
Sex, male	47 (50.5)	22 (48.9)	25 (52.1)
Age, years	67.6 ± 11.6	66.5 ± 12.4	68.7 ± 10.7
Weight, kg	66.0 ± 15.6	67.7 ± 18.0	64.4 ± 13.1
Body mass index, kg/m^2^	25.5 ± 4.3	25.7 ± 4.7	25.4 ± 4.0
Nighttime home (brachial) SBP/DBP, mmHg	132.4 ± 10.4/78.4 ± 7.4	133.7 ± 12.1/79.9 ± 7.4	131.1 ± 8.4/76.9 ± 7.1
Nighttime home (wrist) SBP/DBP, mmHg	130.4 ± 14.5/73.1 ± 9.6	131.3 ± 15.7/73.9 ± 9.7	129.6 ± 13.4/72.3 ± 9.5
Morning home SBP/DBP, mmHg	143.8 ± 13.3/86.7 ± 9.8	144.8 ± 14.9/88.8 ± 10.8	142.9 ± 11.8/84.6 ± 8.2
Bedtime home SBP/DBP, mmHg	135.1 ± 13.6/80.5 ± 9.8	135.9 ± 15.2/82.3 ± 10.7	134.4 ± 12.1/78.8 ± 8.7
Office SBP/DBP, mmHg	147.2 ± 17.8/84.1 ± 12.3	145.9 ± 20.1/84.8 ± 12.8	148.5 ± 15.5/83.4 ± 11.8
Duration of hypertension, months	140.2 ± 122.1	157.4 ± 133.9	125.4 ± 110.3
PAC, pg/ml	39.4 ± 36.8	28.5 ± 23.2	49.7 ± 43.8
PRA, ng/ml/h	2.7 ± 5.8	4.2 ± 8.0	1.3 ± 1.4
Serum potassium, mEq/l	4.0 ± 0.4	4.2 ± 0.4	3.9 ± 0.4
eGFRcreat, ml/min/1.73 m^2^	69.7 ± 17.1	66.1 ± 15.9	73.1 ± 17.6
<30	0	0	0
30–<60	30 (32.3)	19 (42.2)	11 (22.9)
≥60	63 (67.7)	26 (57.8)	37 (77.1)
UACR, mg/gCr	110.4 ± 321.5	121.4 ± 302.5	100.1 ± 341.1
<30	65 (69.9)	29 (64.4)	36 (75.0)
30–<300	19 (20.4)	11 (24.4)	8 (16.7)
≥300	9 (9.7)	5 (11.1)	4 (8.3)
NT-proBNP, pg/ml	83.7 ± 92.3	79.6 ± 75.3	87.4 ± 106.5
Other complications			
Type 2 DM	33 (35.5)	23 (51.1)	10 (20.8)
Diabetic retinopathy^a^	8 (8.6)	7 (15.6)	1 (2.1)
Dyslipidemia	52 (55.9)	27 (60.0)	25 (52.1)
Hyperuricemia	17 (18.3)	9 (20.0)	8 (16.7)
Heart failure	1 (1.1)	0	1 (2.1)
Smoking	14 (15.1)	7 (15.6)	7 (14.6)
Dose of esaxerenone at the end of treatment (last dose)			
1.25 mg	14 (15.1)	13 (28.9)	1 (2.1)
2.5 mg	45 (48.4)	17 (37.8)	28 (58.3)
5 mg	34 (36.6)	15 (33.3)	19 (39.6)

Data are *n* (%) or mean ± SD

*ARB* angiotensin receptor blocker, *CCB* calcium-channel blocker, *DBP* diastolic blood pressure, *DM* diabetes mellitus, *eGFRcreat* creatinine-based estimate of the glomerular filtration rate, *NT-proBNP* N-terminal pro-brain natriuretic peptide, *PAC* plasma aldosterone concentration, *PRA* plasma renin activity, *SBP* systolic blood pressure, *UACR* urinary albumin-to-creatinine ratio

^a^Based on the Davis classification; included patients diagnosed with simple retinopathy, pre-proliferative retinopathy, or proliferative retinopathy

In the total population, the final doses of esaxerenone were 1.25, 2.5, and 5 mg in 15.1%, 48.4%, and 36.6% of the patients, respectively (Table 1). In the ARB subcohort, the final dose of esaxerenone tended to be similarly distributed among the study patients. In the CCB subcohort, most patients had a final esaxerenone dose of 2.5 mg (58.3%), followed by 5 mg (39.6%); only 2.1% of patients had a final dose of 1.25 mg.

### Efficacy

#### BP-lowering effects of esaxerenone

**Fig. 1:**
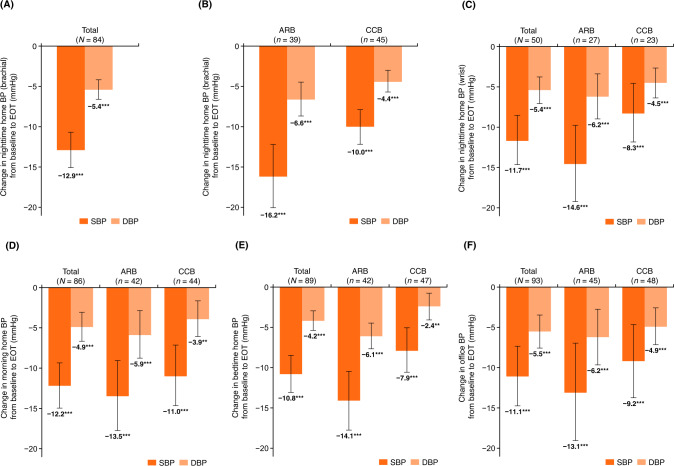
Change from baseline to EOT in nighttime home BP measured using a brachial device (**A**, **B**) or wrist device (**C**), and change in morning home BP (**D**), bedtime home BP (**E**), office BP (**F**) in the total population and ARB or CCB subcohorts (full analysis set). Data are mean (95% confidence interval). **p < 0.01, ***p < 0.001 vs. baseline, paired *t*-test. ARB angiotensin receptor blocker, BP blood pressure, CCB calcium-channel blocker, DBP diastolic blood pressure, EOT end of treatment, SBP systolic blood pressure

A statistically significant decrease in nighttime home BP levels from baseline to EOT measured using the brachial device was shown in the total population (−12.9/−5.4 mmHg, *p* < 0.001) and ARB and CCB subcohorts (−16.2/−6.6 mmHg and −10.0/−4.4 mmHg, respectively; each *p* < 0.001) (Fig. 1A, B). Nighttime home BP, measured using the wrist device, also showed a significant reduction in the total population and ARB and CCB subcohorts (Fig. 1C). Similar significant changes were shown in morning home BP, bedtime home BP, and office BP from baseline to EOT in the total population and ARB and CCB subcohorts (Fig. 1D–F).

**Fig. 2:**
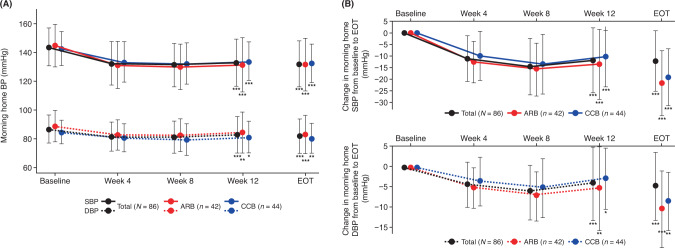
Time course changes (**A**) and change from baseline (**B**) in morning home BP throughout the study period in the total population and ARB or CCB subcohorts (full analysis set). Data are mean ± SD. *p < 0.05, **p < 0.01, ***p < 0.001 vs. baseline, paired *t*-test. ARB angiotensin receptor blocker, BP blood pressure, CCB calcium-channel blocker, DBP diastolic blood pressure, EOT end of treatment, SBP systolic blood pressure

Time course changes in morning home BP, bedtime home BP, and office BP in the total population and ARB or CCB subcohorts are shown in Fig. 2 and Supplementary Fig. 2. Morning home SBP/DBP decreased incrementally until week 4; this reduction in BP was maintained until week 12 and EOT in the total population and ARB or CCB subcohorts (Fig. 2 and Supplementary Table 2). Similar decreasing trends were also observed in bedtime home and office BP (Supplementary Fig. 2 and Supplementary Table 2). In the PPS, similar results were obtained (Supplementary Table 3). Among the total population, the proportion of patients who achieved target BP levels at week 12 in the FAS were 17.2% for nighttime home BP (brachial), 30.1% for nighttime home BP (wrist), 9.7% for morning home BP, and 25.8% for both bedtime home and office BP (Supplementary Table 4). Similar findings were observed in the ARB and CCB subcohorts. The achievement rate of target BP levels in the PPS was similar to that in the FAS (Supplementary Table 5).

#### Effect of esaxerenone on biomarkers

**Fig. 3:**
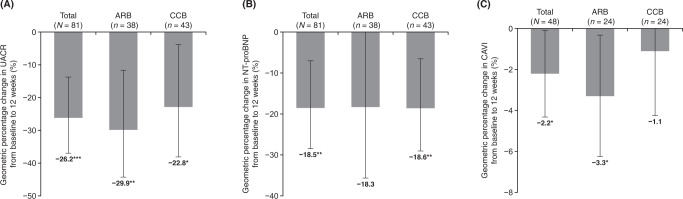
Geometric percentage change in UACR (**A**), NT-proBNP (**B**), and CAVI (**C**) from baseline to 12 weeks (full analysis set). Data are mean (95% confidence interval). *p < 0.05, **p < 0.01, ***p < 0.001 vs. baseline, paired *t*-test. ARB angiotensin receptor blocker, CAVI cardio-ankle vascular index, CCB calcium-channel blocker, CI confidence interval, NT-proBNP N-terminal pro-brain natriuretic peptide, UACR urinary albumin-to-creatinine ratio

The change and the geometric percentage change in UACR from baseline to week 12 in the FAS are shown in Supplementary Table 6 and Fig. 3A. UACR decreased significantly in the total population (geometric percentage change from baseline to week 12, −26.2%; *p* < 0.001). UACR also decreased significantly in each subcohort; the geometric percentage change from baseline to week 12 was −29.9% (*p* < 0.01) and −22.8% (*p* < 0.05) in the ARB and CCB subcohorts, respectively. The change and the geometric percentage change in NT-proBNP from baseline to week 12 in the FAS is shown in Supplementary Table 6 and Fig. 3B. NT-proBNP decreased significantly in the total and CCB subcohort (geometric percentage change from baseline to week 12, −18.5% and −18.6%, respectively [both *p* < 0.01]); in the ARB subcohort, the geometric percentage change decreased by −18.3%, but the change was not statistically significant. PAC and PRA increased with esaxerenone administration from baseline to week 12 in the total population (mean ± SD change: PAC, 47.3 ± 55.3 pg/ml; PRA, 3.8 ± 9.4 ng/ml/h). Similar increases were observed in the ARB and CCB subcohorts. The results for these biomarkers in the PPS are shown in Supplementary Table 7, and the trends of change were similar in the FAS and PPS.

#### Effect of esaxerenone on CAVI

The change and geometric percentage change in CAVI from baseline to week 12 are shown in Supplementary Table 8 and Fig. 3C, and the results for the PPS are shown in Supplementary Table 9. CAVI decreased significantly in the total population and ARB subcohort (geometric percentage change from baseline to week 12, −2.2% and −3.3%, respectively [both *p* < 0.05]); in the CCB subcohort, the geometric percentage change decreased by −1.1%, but the change was not statistically significant. A similar trend was observed in the PPS.

### Safety

TEAEs were reported in 39 (38.6%) patients (Table 2), of whom three (3.0%) discontinued the study treatment. Most TEAEs were mild or moderate. Serious TEAEs were reported in one (1.0%) patient, and the event (pyelonephritis acute) was not related to esaxerenone treatment. Drug-related TEAEs were reported in 17 (16.8%) patients, of whom three (3.0%) discontinued the study treatment. Serious drug-related TEAEs were not reported. The most frequent TEAEs and drug-related TEAEs were associated with serum potassium elevation; for TEAEs, hyperkalemia and blood potassium increased occurred in 11 (10.9%) and four (4.0%) patients, respectively; for drug-related TEAEs, hyperkalemia and blood potassium increased occurred in 10 (9.9%) and three (3.0%) patients, respectively. Among the patients with hyperkalemia and blood potassium increased, one patient each discontinued the study. No cardiovascular-related AEs or deaths were reported during the treatment period.

**Table 2:** Summary of TEAEs (safety analysis set)

	Total *N* = 101	ARB *n* = 48	CCB *n* = 53
Any TEAEs	39 (38.6)	20 (41.7)	19 (35.8)
Drug-related TEAEs	17 (16.8)	9 (18.8)	8 (15.1)
Serious TEAEs	1 (1.0)	1 (2.1)^a^	0
Drug-related serious TEAEs	0	0	0
Discontinued study treatment due to TEAEs	3 (3.0)	3 (6.3)	0
Discontinued study treatment due to drug-related TEAEs	3 (3.0)	3 (6.3)	0
Death	0	0	0
TEAEs occurring in ≥2 patients			
Hyperkalemia	11 (10.9)	4 (8.3)	7 (13.2)
Blood potassium increased	4 (4.0)	3 (6.3)	1 (1.9)
Arthropod sting	3 (3.0)	1 (2.1)	2 (3.8)
Pyrexia	3 (3.0)	1 (2.1)	2 (3.8)
Dizziness	2 (2.0)	0	2 (3.8)
Gastroenteritis	2 (2.0)	1 (2.1)	1 (1.9)
Myalgia	2 (2.0)	2 (4.2)	0
Renal disorder	2 (2.0)	2 (4.2)	0
Drug-related TEAEs occurring in ≥2 patients			
Hyperkalemia	10 (9.9)	4 (8.3)	6 (11.3)
Blood potassium increased	3 (3.0)	3 (6.3)	0
Renal disorder	2 (2.0)	2 (4.2)	0

Data are *n* (%). MedDRA/J version 24.1

*ARB* angiotensin receptor blocker, *CCB* calcium-channel blocker, *TEAE* treatment emergent adverse event

^a^Pyelonephritis acute

**Fig. 4:**
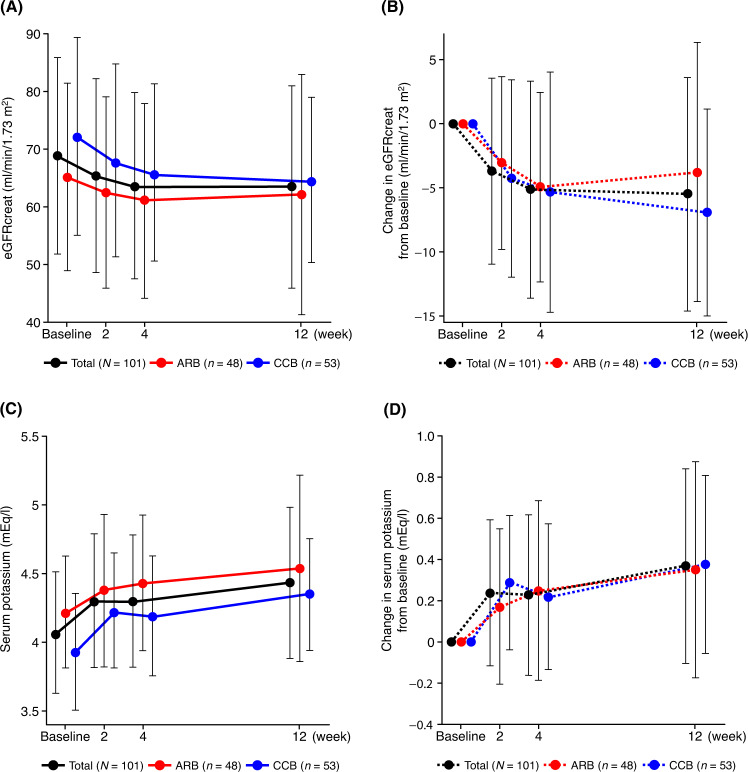
Time course changes and change from baseline in eGFRcreat (**A**, **B**) and serum potassium (**C**, **D**) during the study period in the total population and ARB or CCB subcohorts (safety analysis set). Data are mean ± SD. ARB angiotensin receptor blocker, CCB calcium-channel blocker, eGFRcreat creatinine-based estimated glomerular filtration rate

eGFRcreat initially decreased over the first 4 weeks, remaining constant thereafter (Fig. 4A). The mean change from baseline to week 12 in eGFRcreat was −5.5 ± 9.1 ml/min/1.73 m^2^ (Fig. 4B). A similar trend of reduction in eGFRcreat was observed in the ARB and CCB subcohorts (mean changes from baseline to week 12 of −3.8 ± 10.1 and −6.9 ± 8.1 ml/min/1.73 m^2^, respectively).

Serum potassium levels tended to increase during the study period. The increase was greater in the first 2 weeks and slower thereafter (Fig. 4C). Serum potassium increased by 0.24 mEq/l at 2 weeks and 0.37 mEq/l at 12 weeks (Fig. 4D). There was no notable difference between the ARB and CCB subcohorts.

Eleven (10.9%) patients had serum potassium levels ≥5.5 mEq/l during the study period, including nine (18.8%) patients in the ARB subcohort and two (3.8%) patients in the CCB subcohort (Supplementary Table 10). Two (2.0%) patients had serum potassium ≥6.0 mEq/l, both in the ARB subcohort (4.2%), and discontinued the study.

## Discussion

In the EARLY-NH study, two newly developed brachial and wrist HBPM devices were used to demonstrate the efficacy of esaxerenone in significantly reducing nighttime home BP in patients with uncontrolled nocturnal hypertension despite treatment with an ARB or CCB.

### BP-lowering effects

Esaxerenone significantly reduced the primary endpoint of nighttime home BP (brachial) from baseline to week 12 in the total population (−12.9/−5.4 mmHg, *p* < 0.001) and in the ARB and CCB subcohorts (−16.2/−6.6 mmHg and −10.0/−4.4 mmHg, respectively; all *p* < 0.001). In the previous long-term phase 3 study, esaxerenone reduced nighttime BP from baseline to week 12 by −14.3/−8.5 mmHg in combination with an ARB, and −11.5/−5.6 mmHg with a CCB (unpublished data), which are consistent with those of the present EARLY-NH study. Furthermore, in the ESAX-HTN study, which evaluated the efficacy of esaxerenone monotherapy, the changes in nighttime BP after 12 weeks of treatment were −9.9/−4.4 mmHg at 2.5 mg and −13.7/−6.5 mmHg at 5 mg [23]. Considering the final dose ratio in the present study (2.5 mg: 48.4%, 5 mg: 36.6%), the results are also considered to be similar to those of this study. Although the definition of nighttime BP in these phase 3 studies (hourly arithmetic mean values from 22:00 to 6:00 h, measured with ABPM) differs from the definition in this study, these results suggest that esaxerenone would exert a consistent favorable effect on nocturnal hypertension. In addition, esaxerenone has been shown to improve dipping pattern and reduce riser and non-dipper types [15], and it is possible that the dipping patterns of the patients were also improved in this study.

Morning home, bedtime home, and office BP appeared to show an upward trend from week 8 to week 12. At week 8, BP was measured only in patients who received a dose increase at week 4, and at week 12, BP was measured in both patients who received the dose increase at week 4 and those who did not. Thus, the appearance of an upward trend in BP from week 8 to week 12 was presumably because of a smaller decrease in BP in the patients who did not increase their dose of esaxerenone at week 4.

The antihypertensive effect of esaxerenone depends on patient background. A subgroup analysis of the ESAX-HTN study showed that in patients with essential hypertension, decreases in office BP were numerically greater in female patients, patients aged ≥65 years, patients with low body mass index (<25 kg/m^2^), and patients with lower baseline levels of PAC (<120 pg/ml) and PRA (<1.0 ng/ml/h) [24]. In a multivariate analysis of seven phase 3 studies integrating hypertensive patients with type 2 DM, moderate renal dysfunction, and primary aldosteronism in addition to essential hypertension, factors such as female sex, lower body weight, lower PAC, and lower UACR were significantly associated with a stronger reduction in office BP with esaxerenone treatment [25]. A subgroup analysis of this EARLY-NH study showed a similar trend to the previous results, with numerically greater reductions in office BP in patients aged ≥65 years, with low body mass index (<25 kg/m^2^), and with low baseline PRA levels (<1.0 ng/ml/h). However, regarding nighttime home BP (brachial), no clear numerical difference was observed (data not shown). Additionally, in the present study, the antihypertensive effect of esaxerenone on each BP, including nighttime BP, appeared to be numerically stronger in the ARB subcohort compared with the CCB subcohort. Although we did not make statistical comparisons between the subcohorts as this was not specified in the study design, this difference may also be partly due to patient background: The ARB subcohort had a higher complication rate of DM (51.1% vs. 20.8%) and moderate renal dysfunction (42.2% vs. 22.9%) than the CCB subcohort. Affected by the difference in these patient background characteristics, the final dose of esaxerenone also differed between the ARB and CCB subcohorts (ARB: 1.25 mg, 28.9%; 2.5 mg, 37.8%; and CCB: 1.25 mg, 2.1%; 2.5 mg, 58.3%). Further analysis is needed to determine which patient background characteristics affect the nighttime BP-lowering effect of esaxerenone.

Risk factors for nocturnal hypertension include increased vascular resistance and arterial stiffness, excessive salt intake, and salt sensitivity, while salt sensitivity is also enhanced by renal impairment, sympathetic hyperactivity, and activation of the renin-angiotensin-aldosterone system (RAS) [2]. Among these factors, sympathetic hyperactivity and RAS activation cause elevated plasma aldosterone levels and activate MRs in a ligand-dependent manner, whereas excessive salt intake and salt sensitivity activate MRs in a ligand-independent manner via MR-interacting factors such as Rac1 [13]. Thus, because MRs are activated in the presence of risk factors for nocturnal hypertension, MRBs are considered an optimal antihypertensive drug class capable of controlling nocturnal hypertension. The MRBs spironolactone and eplerenone have also been reported to reduce nocturnal hypertension [26–28], and esaxerenone is assumed to exert a more sustained and potent nighttime BP-lowering effect among these MRBs. This is because its half-life (18.6–25.1 h) is longer than that of eplerenone and spironolactone (3–5 and >12 h, respectively), and its MR binding affinities are 76-fold and 4-fold stronger, respectively [29]. Indeed, its superiority for 24-h BP reduction over eplerenone was verified in the ESAX-HTN study [24]. In this study, esaxerenone significantly reduced not only nighttime BP but also morning home, bedtime home, and office BP, suggesting that it exerts a sustained antihypertensive effect over 24 h, which may be due to its pharmacological profile.

### Organ-protective effects

Esaxerenone has demonstrated a UACR-lowering effect in phase 3 studies [16–18] and a post-marketing clinical study [19]. In the EARLY-NH study, the patient ratios at baseline in each UACR category of normoalbuminuria (<30 mg/gCr), microalbuminuria (30 to <300 mg/gCr), and macroalbuminuria (≥300 mg/gCr) were 69.9%, 20.4% and 9.7%, respectively, and only about 30% of patients had albuminuria. In that study, 12 weeks of esaxerenone treatment significantly reduced UACR by −26.2% in the total population. Because esaxerenone continuously reduces UACR up to 6 months after administration, followed by a steady state [17], further UACR reduction can be expected with long-term administration. Although part of this UACR-lowering effect is due to the reduction in circulating and renal blood flow by lowering BP [19], recent statistical medication analysis has shown that direct MR inhibition, independent of the antihypertensive effect, reduces UACR [30].

Esaxerenone significantly reduced NT-proBNP by −18.5% in the total population. The J-HOP study suggested that NT-proBNP levels predict organ damage derived from elevated nighttime BP [8, 31, 32]. In a post hoc subanalysis to examine the effects of esaxerenone on nighttime BP and NT-proBNP levels stratified by dipping pattern, esaxerenone significantly reduced NT-proBNP from baseline to week 28 in risers, non-dippers, dippers, and extreme dippers, and increased the percentage of patients with normal NT-proBNP levels (<55 pg/ml) in risers from 48.0% to 75.0% [15].

In this study, esaxerenone reduced CAVI by −2.2% in the total population, and −3.3% and −1.1% in the ARB and CCB subcohorts, respectively, with 12 weeks of treatment. The vasoprotective effects of eplerenone and spironolactone have been previously reported [26, 33], but this is the first report of esaxerenone improving CAVI.

Albuminuria is a risk factor for renal and cardiac events [34–36], NT-proBNP is a predictor of cardiovascular risk and kidney organ damage [37–39], and CAVI is a risk factor for cardiovascular events [40, 41]. Based on the improvement of these parameters demonstrated in this study, esaxerenone is expected to be an effective treatment option to reduce the risk of renal and cardiovascular events in nocturnal hypertensive patients with a high risk of cardiovascular events.

### Effect on serum potassium

Serum potassium elevation, a well-known adverse effect of MRBs [42] was the most frequent TEAE and drug-related TEAE in the study, although the severity of these events was mild or moderate: hyperkalemia, with TEAEs in 11 (10.9%) patients and drug-related TEAEs in 10 (9.9%) patients; blood potassium increased, with TEAEs in four (4.0%) patients and drug-related TEAEs in three (3.0%) patients. From the previous phase 3 studies, the following factors were associated with a risk of serum potassium elevation during treatment with esaxerenone: DM with albuminuria or proteinuria, moderate renal dysfunction (eGFR ≥30 to <60 ml/min/1.73 m^2^, respectively), being elderly, patients with higher baseline serum potassium levels, and patients receiving RAS inhibitors [42]. These factors are listed as potential risks in the esaxerenone package insert [20].

Of the 13 patients with drug-related TEAEs related to serum potassium elevation, nine were ≥80 years, nine had eGFR ≤60 ml/min/1.73 m^2^, and seven met both criteria. Of the 11 patients with serum potassium levels ≥5.5 mEq/l, six had onset during the summer in Japan (July to mid-September), and nine were in the ARB subcohort. These results indicate that esaxerenone, to avoid serum potassium elevation, should be administered with caution to patients ≥80 years, with moderate renal dysfunction (eGFRcreat ≤60 ml/min/1.73 m^2^), and with concomitant ARBs. In addition, elderly patients are more likely to become dehydrated during the summer months, indicating that more caution is needed for this population.

The 10.9% incidence of serum potassium level ≥5.5 mEq/l during the study period was slightly higher than that reported in phase 3 studies of patients with essential hypertension (3.0–6.3%) [42], and the incidence of serum potassium level ≥6.0 mEq/l was similar (0.5–0.6%) to that reported previously [14, 24]. The higher incidence of ≥5.5 mEq/l in this study may be explained by serum potassium elevation in patients ≥80 years with moderate renal dysfunction.

### Other safety measures

eGFRcreat decreased by the first 4 weeks of treatment and remained constant thereafter in the total population and both subcohorts. eGFRcreat changes at weeks 4–12 ranged from −5.1 to −5.5 ml/min/1.73 m^2^, which is similar to previous studies of esaxerenone [14, 16–19, 24]. The trends in eGFRcreat and BP change were consistent, suggesting that the eGFR decline in the early phase of treatment was associated with normalization of glomerular hyperfiltration due to reduced renal blood flow. Thus, no clinical safety concerns were raised regarding transient reduction in eGFRcreat.

The incidences of TEAEs, other than serum potassium elevation, were similar to those reported in previous studies [14, 16–19, 24], and no new safety concerns were identified. Our results suggest that esaxerenone can be used safely, although caution should be paid to serum potassium elevation.

### Nighttime BP-lowering effect evaluated by two nocturnal HBPM devices

Nighttime BP was measured with two device models: brachial and wrist, and BP measurements showed a trend toward lower BP values and larger SDs with the wrist type compared to the brachial type (SBP/DBP at baseline, 132.4 ± 10.4/78.4 ± 7.4 for the brachial type and 130.4 ± 14.5/73.1 ± 9.6 for the wrist type). This trend was also observed at each measurement time point. However, the “change in BP” had similar BP values and SDs for the brachial and the wrist devices (SBP/DBP at EOT, −12.9 ± 10.3/−5.4 ± 5.6 for the brachial, and −11.7 ± 10.9/−5.4 ± 6.0 for the wrist). As previously reported [4–7], these results suggest that the wrist device can be used to monitor nighttime BP changes as well as the brachial type recommended in the guidelines. Advances in the development of HBPM devices reduce patients’ burdens, and enable them to monitor nighttime BP, which has been previously only measurable by ABPM. In this setting, drugs like esaxerenone, which improve nocturnal hypertension and mitigate risk factors for organ damage and cardiovascular events, become important.

### Limitations

The present study has some limitations, including the small sample size, which may have led to insufficient statistical power. The study had a single-arm design that did not include a placebo. However, previous placebo-controlled esaxerenone clinical trials have confirmed the significant antihypertensive action of the drug [17, 43]. Future clinical studies with a comparative study design are warranted. More than 90% of the patients in this study were enrolled between April and September and so received esaxerenone during a relatively warm period in the Northern Hemisphere. Therefore, the overall trend in BP changes in this study should be considered as a result obtained during the warmer months. As this study was conducted in Japan, the generalizability of the results to other populations should be interpreted with caution.

### Perspectives in Asia

Asians are more likely to have factors relating to salt-sensitive gene polymorphism of the RAS, resulting in a higher prevalence of nocturnal hypertension [44, 45]. The favorable nighttime BP-lowering effect of esaxerenone is expected to contribute to achieving the goal of “zero” cardiovascular events in Asia [44–46].

## Conclusions

In patients with uncontrolled nocturnal hypertension being treated with an ARB or CCB, esaxerenone demonstrated a 24-h sustained antihypertensive effect beyond nocturnal hypertension by lowering nighttime BP, morning and bedtime home BP, and office BP. Furthermore, esaxerenone also demonstrated organ-protective effects by reducing albuminuria, NT-proBNP, and CAVI. Although careful monitoring of serum potassium is required for patients ≥80 years and with moderate renal dysfunction, clinically problematic reductions in eGFRcreat were not observed, and esaxerenone can be safely used in this population. Esaxerenone may not only be an optimal treatment option for patients with nocturnal hypertension, but may also be useful in the total BP management.

## Supplementary information

Supplementary Tables

Supplementary Figures

Supplementary Figure legends

## Data Availability

The anonymized data underlying the results presented in this manuscript may be made available to researchers upon submission of a reasonable request to the corresponding author. The decision to disclose the data will be made by the corresponding author and the funder, Daiichi Sankyo Co., Ltd. Data disclosure can be requested for 36 months from article publication.
