# Supplementary material for: Nighttime home blood pressure lowering effect of esaxerenone in patients with uncontrolled nocturnal hypertension: the EARLY-NH study
Source: Hypertens Res. 2023 May 12;46(7):1782–94. doi: 10.1038/s41440-023-01292-0 (PMC10319630; doi:10.1038/s41440-023-01292-0)
Supplement: Supplementary file 1 — Supplementary Tables [file 41440_2023_1292_MOESM1_ESM.docx]

**Supplementary Tables**

## Supplementary Table 1 List of participating institutions and representative physicians^a^

| **Institution** | **Representative physicians** |
| --- | --- |
| Division of Cardiovascular Medicine, Department of Medicine, Jichi Medical University School of Medicine | Kazuomi Kario (principal investigator) |
| Department of Cardiology, Hiroshima City Asa Hospital | Michiaki Nagai |
| Hosoda Clinic | Masaki Akahata |
| Ikeda Clinic | Kazuo Ikeda |
| Kan-etsu Central Hospital^b^ | Yawara Niijima, Toshiaki Ando |
| Kashinoki Internal Medicine | Hajime Ishii |
| Kato Clinic of Internal Medicine | Mitsutoshi Kato |
| Katsuya Clinic | Tomohiro Katsuya |
| Minamisanriku Hospital | Masafumi Nishizawa |
| Okuda Clinic | Takeshi Okuda |
| Department of Cardiovascular Medicine, Onga Nakama Medical Association Onga Hospital | Tetsuro Yoshida |
| Shiraiwa Medical Clinic | Toshihiko Shiraiwa |
| Takahashi Family Clinic | Nobuo Takahashi |
| Tsuruma Kaneshiro Diabetes Clinic | Taro Asakura |
| Uchiyama Clinic | Kazuaki Uchiyama |
| Washiya Hospital | Toshimitsu Kitajima |
| Yamagiwa Clinic | Kayo Yamagiwa |

^a^Institutions are listed in alphabetical order except for Jichi Medical University School of Medicine.

^b^Facilities where there was a change in the principal investigator during the course of the study.

## Supplementary Table 2 Change in BP from baseline (full analysis set)

|  | **Total**  ***N* = 93** | | **ARB subcohort**  ***n* = 45** | | **CCB subcohort**  ***n* = 48** | |
| --- | --- | --- | --- | --- | --- | --- |
|  | ***n*** | **Mean ± SD** | ***n*** | **Mean ± SD** | **n** | **Mean ± SD** |
| **Nighttime home SBP (brachial)** | | | | | | |
| Baseline | 93 | 132.4 ± 10.4 | 45 | 133.7 ± 12.1 | 48 | 131.1 ± 8.4 |
| Week 12 | 81 | 119.6 ± 10.7 | 37 | 118.2 ± 11.2 | 44 | 120.7 ± 10.3 |
| Change from baseline | 81 | −12.8 ± 10.1*** | 37 | −15.8 ± 12.3*** | 44 | −10.3 ± 7.1*** |
| EOT | 84 | 119.4 ± 10.8 | 39 | 117.6 ± 11.2 | 45 | 120.9 ± 10.2 |
| Change from baseline | 84 | −12.9 ± 10.3*** | 39 | −16.2 ± 12.3*** | 45 | −10.0 ± 7.1*** |
| **Nighttime home DBP (brachial)** | | | | | | |
| Baseline | 93 | 78.4 ± 7.4 | 45 | 79.9 ± 7.4 | 48 | 76.9 ± 7.1 |
| Week 12 | 81 | 72.8 ± 7.4 | 37 | 73.7 ± 8.0 | 44 | 72.0 ± 6.8 |
| Change from baseline | 81 | −5.4 ± 5.5*** | 37 | −6.5 ± 6.4*** | 44 | −4.5 ± 4.5*** |
| EOT | 84 | 72.7 ± 7.5 | 39 | 73.2 ± 8.1 | 45 | 72.3 ± 6.9 |
| Change from baseline | 84 | −5.4 ± 5.6*** | 39 | −6.6 ± 6.5*** | 45 | −4.4 ± 4.5*** |
| **Nighttime home SBP (wrist)** | | | | | | |
| Baseline | 74 | 130.4 ± 14.5 | 36 | 131.3 ± 15.7 | 38 | 129.6 ± 13.4 |
| Week 12 | 59 | 117.7 ± 12.7 | 32 | 116.4 ± 14.8 | 27 | 119.4 ± 9.6 |
| Change from baseline | 50 | −11.7 ± 10.9*** | 27 | −14.6 ± 12.0*** | 23 | −8.3 ± 8.4*** |
| EOT | 59 | 117.7 ± 12.7 | 32 | 116.4 ± 14.8 | 27 | 119.4 ± 9.6 |
| Change from baseline | 50 | −11.7 ± 10.9*** | 27 | −14.6 ± 12.0*** | 23 | −8.3 ± 8.4*** |
| **Nighttime home DBP (wrist)** | | | | | | |
| Baseline | 74 | 73.1 ± 9.6 | 36 | 73.9 ± 9.7 | 38 | 72.3 ± 9.5 |
| Week 12 | 59 | 67.7 ± 8.4 | 32 | 68.8 ± 8.4 | 27 | 66.4 ± 8.3 |
| Change from baseline | 50 | −5.4 ± 6.0*** | 27 | −6.2 ± 7.0*** | 23 | −4.5 ± 4.3*** |
| EOT | 59 | 67.7 ± 8.4 | 32 | 68.8 ± 8.4 | 27 | 66.4 ± 8.3 |
| Change from baseline | 50 | −5.4 ± 6.0*** | 27 | −6.2 ± 7.0*** | 23 | −4.5 ± 4.3*** |
| **Morning home SBP** | | | | | | |
| Baseline | 86 | 143.8 ± 13.3 | 42 | 144.8 ± 14.9 | 44 | 142.9 ± 11.8 |
| Week 4 | 82 | 132.5 ± 15.2 | 38 | 131.3 ± 16.3 | 44 | 133.5 ± 14.2 |
| Change from baseline | 77 | −11.2 ± 9.9 | 36 | −12.6 ± 8.9 | 41 | −9.9 ± 10.6 |
| Week 8 | 64 | 131.3 ± 15.0 | 34 | 130.2 ± 15.7 | 30 | 132.4 ± 14.3 |
| Change from baseline | 60 | −14.6 ± 12.2 | 32 | −15.5 ± 11.7 | 28 | −13.5 ± 12.9 |
| Week 12 | 68 | 132.8 ± 16.2 | 33 | 131.5 ± 18.8 | 35 | 133.9 ± 13.5 |
| Change from baseline | 64 | −11.9 ± 14.0*** | 31 | −13.5 ± 15.0*** | 33 | −10.3 ± 13.0*** |
| EOT | 91 | 132.1 ± 15.8 | 44 | 131.6 ± 18.4 | 47 | 132.5 ± 13.3 |
| Change from baseline | 86 | −12.2 ± 13.1*** | 42 | −13.5 ± 14.0*** | 44 | −11.0 ± 12.2*** |
| **Morning home DBP** | | | | | | |
| Baseline | 86 | 86.7 ± 9.8 | 42 | 88.8 ± 10.8 | 44 | 84.6 ± 8.2 |
| Week 4 | 82 | 81.6 ± 10.1 | 38 | 82.7 ± 10.5 | 44 | 80.6 ± 9.8 |
| Change from baseline | 77 | −4.5 ± 5.6 | 36 | −5.4 ± 4.9 | 41 | −3.7 ± 6.0 |
| Week 8 | 64 | 81.1 ± 11.3 | 34 | 82.5 ± 11.5 | 30 | 79.5 ± 11.0 |
| Change from baseline | 60 | −6.4 ± 6.7 | 32 | −7.3 ± 6.1 | 28 | −5.3 ± 7.3 |
| Week 12 | 68 | 82.9 ± 12.6 | 33 | 84.5 ± 14.2 | 35 | 81.3 ± 10.9 |
| Change from baseline | 64 | −4.2 ± 9.1*** | 31 | −5.5 ± 10.5** | 33 | −3.0 ± 7.6* |
| EOT | 91 | 81.6 ± 11.9 | 44 | 83.0 ± 13.4 | 47 | 80.4 ± 10.4 |
| Change from baseline | 86 | −4.9 ± 8.4*** | 42 | −5.9 ± 9.5*** | 44 | −3.9 ± 7.2** |
| **Bedtime home SBP** | | | | | | |
| Baseline | 89 | 135.1 ± 13.6 | 42 | 135.9 ± 15.2 | 47 | 134.4 ± 12.1 |
| Week 4 | 82 | 125.8 ± 15.0 | 40 | 125.5 ± 18.8 | 42 | 126.0 ± 10.3 |
| Change from baseline | 79 | −9.5 ± 10.6 | 38 | −11.3 ± 11.5 | 41 | −7.7 ± 9.4 |
| Week 8 | 64 | 123.0 ± 14.4 | 33 | 120.9 ± 16.9 | 31 | 125.4 ± 11.0 |
| Change from baseline | 61 | −11.9 ± 10.9 | 31 | −14.7 ± 11.8 | 30 | −8.9 ± 9.2 |
| Week 12 | 70 | 124.4 ± 15.3 | 35 | 123.9 ± 18.6 | 35 | 124.9 ± 11.3 |
| Change from baseline | 68 | −10.6 ± 10.7*** | 33 | −12.9 ± 11.0*** | 35 | −8.3 ± 10.1*** |
| EOT | 93 | 124.8 ± 15.2 | 45 | 123.0 ± 17.8 | 48 | 126.6 ± 12.2 |
| Change from baseline | 89 | −10.8 ± 11.0*** | 42 | −14.1 ± 11.7*** | 47 | −7.9 ± 9.5*** |
| **Bedtime home DBP** | | | | | | |
| Baseline | 89 | 80.5 ± 9.8 | 42 | 82.3 ± 10.7 | 47 | 78.8 ± 8.7 |
| Week 4 | 82 | 77.2 ± 9.8 | 40 | 77.8 ± 11.1 | 42 | 76.5 ± 8.3 |
| Change from baseline | 79 | −3.4 ± 6.0 | 38 | −5.0 ± 6.0 | 41 | −1.9 ± 5.7 |
| Week 8 | 64 | 75.4 ± 10.7 | 33 | 75.6 ± 12.4 | 31 | 75.2 ± 8.7 |
| Change from baseline | 61 | −4.7 ± 6.1 | 31 | −6.4 ± 6.2 | 30 | −3.0 ± 5.6 |
| Week 12 | 70 | 76.5 ± 10.8 | 35 | 77.7 ± 12.7 | 35 | 75.3 ± 8.4 |
| Change from baseline | 68 | −4.2 ± 5.9*** | 33 | −5.6 ± 5.2*** | 35 | −2.9 ± 6.4* |
| EOT | 93 | 76.5 ± 10.1 | 45 | 76.7 ± 11.8 | 48 | 76.3 ± 8.3 |
| Change from baseline | 89 | −4.2 ± 5.8*** | 42 | −6.1 ± 5.3*** | 47 | −2.4 ± 5.7** |
| **Office SBP** | | | | | | |
| Baseline | 93 | 147.2 ± 17.8 | 45 | 145.9 ± 20.1 | 48 | 148.5 ± 15.5 |
| Week 4 | 87 | 134.4 ± 16.4 | 41 | 131.2 ± 17.2 | 46 | 137.2 ± 15.3 |
| Change from baseline | 87 | −12.4 ± 17.7 | 41 | −13.9 ± 17.7 | 46 | −11.0 ± 17.7 |
| Week 8 | 70 | 137.3 ± 15.8 | 35 | 132.6 ± 14.0 | 35 | 141.9 ± 16.3 |
| Change from baseline | 70 | −11.1 ± 16.8 | 35 | −13.0 ± 16.1 | 35 | −9.2 ± 17.4 |
| Week 12 | 82 | 136.4 ± 17.1 | 38 | 132.9 ± 17.6 | 44 | 139.5 ± 16.2 |
| Change from baseline | 82 | −10.4 ± 17.9*** | 38 | −12.1 ± 20.1*** | 44 | −9.0 ± 15.7*** |
| EOT | 93 | 136.2 ± 17.2 | 45 | 132.8 ± 17.8 | 48 | 139.3 ± 16.2 |
| Change from baseline | 93 | −11.1 ± 18.0*** | 45 | −13.1 ± 20.2*** | 48 | −9.2 ± 15.6*** |
| **Office DBP** | | | | | | |
| Baseline | 93 | 84.1 ± 12.3 | 45 | 84.8 ± 12.8 | 48 | 83.4 ± 11.8 |
| Week 4 | 87 | 78.4 ± 12.6 | 41 | 78.5 ± 13.1 | 46 | 78.3 ± 12.2 |
| Change from baseline | 87 | −5.5 ± 9.1 | 41 | −6.3 ± 9.8 | 46 | −4.8 ± 8.5 |
| Week 8 | 70 | 79.2 ± 13.0 | 35 | 78.6 ± 11.2 | 35 | 79.7 ± 14.8 |
| Change from baseline | 70 | −5.3 ± 8.1 | 35 | −6.6 ± 8.4 | 35 | −4.0 ± 7.7 |
| Week 12 | 82 | 78.8 ± 12.2 | 38 | 79.3 ± 13.1 | 44 | 78.3 ± 11.4 |
| Change from baseline | 82 | −4.9 ± 9.1*** | 38 | −5.3 ± 10.5** | 44 | −4.5 ± 7.8*** |
| EOT | 93 | 78.5 ± 12.5 | 45 | 78.6 ± 14.0 | 48 | 78.5 ± 11.0 |
| Change from baseline | 93 | −5.5 ± 9.8*** | 45 | −6.2 ± 11.5*** | 48 | −4.9 ± 8.0*** |

**p* < 0.05, ***p* < 0.01, ****p* < 0.001 vs. baseline, paired *t*-test.

*P*-values were not calculated for changes from baseline to Week 4 and Week 8.

*ARB* angiotensin receptor blocker, *BP* blood pressure, *CCB* calcium-channel blocker, *DBP* diastolic blood pressure, *EOT* end of treatment, *SBP* systolic blood pressure.

## Supplementary Table 3 Change in BP from baseline (per-protocol set)

|  | **Total**  ***N* = 89** | | **ARB subcohort**  ***n* = 44** | | **CCB subcohort**  ***n* = 45** | |
| --- | --- | --- | --- | --- | --- | --- |
|  | ***n*** | **Mean ± SD** | ***n*** | **Mean ± SD** | ***n*** | **Mean ± SD** |
| **Nighttime home SBP (brachial)** | | | | | | |
| Baseline | 89 | 132.1 ± 10.3 | 44 | 134.0 ± 12.1 | 45 | 130.3 ± 8.0 |
| Week 12 | 78 | 119.2 ± 10.5 | 36 | 118.4 ± 11.3 | 42 | 119.9 ± 9.8 |
| Change from baseline | 78 | −13.0 ± 10.2*** | 36 | −15.9 ± 12.4*** | 42 | −10.5 ± 7.1*** |
| EOT | 81 | 119.0 ± 10.5 | 38 | 117.8 ± 11.3 | 43 | 120.1 ± 9.7 |
| Change from baseline | 81 | −13.1 ± 10.4*** | 38 | −16.3 ± 12.4*** | 43 | −10.3 ± 7.2*** |
| **Nighttime home DBP (brachial)** | | | | | | |
| Baseline | 89 | 78.3 ± 7.5 | 44 | 80.0 ± 7.5 | 45 | 76.8 ± 7.3 |
| Week 12 | 78 | 72.7 ± 7.5 | 36 | 73.8 ± 8.1 | 42 | 71.8 ± 6.9 |
| Change from baseline | 78 | −5.4 ± 5.6*** | 36 | −6.4 ± 6.5*** | 42 | −4.6 ± 4.6*** |
| EOT | 81 | 72.6 ± 7.5 | 38 | 73.3 ± 8.2 | 43 | 72.0 ± 7.0 |
| Change from baseline | 81 | −5.5 ± 5.7*** | 38 | −6.6 ± 6.6*** | 43 | −4.4 ± 4.6*** |
| **Nighttime home SBP (wrist)** | | | | | | |
| Baseline | 71 | 130.1 ± 14.3 | 35 | 131.7 ± 15.8 | 36 | 128.5 ± 12.7 |
| Week 12 | 57 | 117.9 ± 12.5 | 31 | 117.1 ± 14.5 | 26 | 119.0 ± 9.6 |
| Change from baseline | 49 | −11.4 ± 10.9*** | 26 | −14.3 ± 12.1*** | 23 | −8.3 ± 8.4*** |
| EOT | 57 | 117.9 ± 12.5 | 31 | 117.1 ± 14.5 | 26 | 119.0 ± 9.6 |
| Change from baseline | 49 | −11.4 ± 10.9*** | 26 | −14.3 ± 12.1*** | 23 | −8.3 ± 8.4*** |
| **Nighttime home DBP (wrist)** | | | | | | |
| Baseline | 71 | 73.1 ± 9.8 | 35 | 74.0 ± 9.8 | 36 | 72.3 ± 9.8 |
| Week 12 | 57 | 67.8 ± 8.4 | 31 | 69.1 ± 8.4 | 26 | 66.2 ± 8.4 |
| Change from baseline | 49 | −5.3 ± 5.9*** | 26 | −6.0 ± 7.1*** | 23 | −4.5 ± 4.3*** |
| EOT | 57 | 67.8 ± 8.4 | 31 | 69.1 ± 8.4 | 26 | 66.2 ± 8.4 |
| Change from baseline | 49 | −5.3 ± 5.9*** | 26 | −6.0 ± 7.1*** | 23 | −4.5 ± 4.3*** |
| **Morning home SBP** | | | | | | |
| Baseline | 82 | 143.7 ± 13.4 | 41 | 144.7 ± 15.1 | 41 | 142.7 ± 11.7 |
| Week 4 | 79 | 132.7 ± 15.4 | 37 | 131.4 ± 16.5 | 42 | 133.8 ± 14.5 |
| Change from baseline | 74 | −10.8 ± 9.6 | 35 | −12.4 ± 9.0 | 39 | −9.4 ± 10.0 |
| Week 8 | 61 | 131.4 ± 15.2 | 33 | 130.5 ± 15.9 | 28 | 132.6 ± 14.6 |
| Change from baseline | 57 | −14.3 ± 11.7 | 31 | −15.3 ± 11.8 | 26 | −13.1 ± 11.8 |
| Week 12 | 65 | 133.0 ± 16.5 | 32 | 131.5 ± 19.1 | 33 | 134.4 ± 13.7 |
| Change from baseline | 61 | −11.5 ± 13.7*** | 30 | −13.5 ± 15.3*** | 31 | −9.5 ± 12.0*** |
| EOT | 87 | 132.1 ± 16.1 | 43 | 131.6 ± 18.6 | 44 | 132.6 ± 13.5 |
| Change from baseline | 82 | −12.0 ± 12.9*** | 41 | −13.5 ± 14.2*** | 41 | −10.6 ± 11.5*** |
| **Morning home DBP** | | | | | | |
| Baseline | 82 | 86.9 ± 9.9 | 41 | 88.8 ± 11.0 | 41 | 84.9 ± 8.3 |
| Week 4 | 79 | 81.7 ± 10.2 | 37 | 82.6 ± 10.6 | 42 | 80.9 ± 9.8 |
| Change from baseline | 74 | −4.4 ± 5.5 | 35 | −5.5 ± 5.0 | 39 | −3.4 ± 5.7 |
| Week 8 | 61 | 81.4 ± 11.3 | 33 | 82.6 ± 11.6 | 28 | 80.0 ± 11.1 |
| Change from baseline | 57 | −6.2 ± 6.5 | 31 | −7.2 ± 6.2 | 26 | −5.0 ± 6.8 |
| Week 12 | 65 | 83.1 ± 12.7 | 32 | 84.4 ± 14.4 | 33 | 81.8 ± 10.9 |
| Change from baseline | 61 | −4.0 ± 9.1*** | 30 | −5.6 ± 10.6** | 31 | −2.5 ± 7.1 |
| EOT | 87 | 81.9 ± 12.0 | 43 | 82.9 ± 13.5 | 44 | 80.8 ± 10.4 |
| Change from baseline | 82 | −4.8 ± 8.4*** | 41 | −6.0 ± 9.6*** | 41 | −3.7 ± 6.8** |
| **Bedtime home SBP** | | | | | | |
| Baseline | 85 | 134.9 ± 13.6 | 41 | 136.4 ± 15.1 | 44 | 133.5 ± 11.9 |
| Week 4 | 79 | 126.0 ± 15.1 | 39 | 125.8 ± 19.0 | 40 | 126.2 ± 10.4 |
| Change from baseline | 76 | −9.2 ± 10.4 | 37 | −11.5 ± 11.7 | 39 | −7.1 ± 8.8 |
| Week 8 | 61 | 123.1 ± 14.5 | 32 | 121.3 ± 17.0 | 29 | 125.0 ± 11.1 |
| Change from baseline | 58 | −11.8 ± 11.0 | 30 | −14.9 ± 12.0 | 28 | −8.5 ± 8.9 |
| Week 12 | 67 | 124.3 ± 15.4 | 34 | 124.3 ± 18.8 | 33 | 124.3 ± 11.3 |
| Change from baseline | 65 | −10.7 ± 10.9*** | 32 | −13.1 ± 11.1*** | 33 | −8.3 ± 10.3*** |
| EOT | 89 | 124.4 ± 15.1 | 44 | 123.2 ± 17.9 | 45 | 125.6 ± 11.7 |
| Change from baseline | 85 | −11.0 ± 11.1*** | 41 | −14.3 ± 11.8*** | 44 | −8.0 ± 9.7*** |
| **Bedtime home DBP** | | | | | | |
| Baseline | 85 | 80.6 ± 10.0 | 41 | 82.5 ± 10.7 | 44 | 78.8 ± 9.0 |
| Week 4 | 79 | 77.4 ± 9.7 | 39 | 78.0 ± 11.2 | 40 | 76.8 ± 8.2 |
| Change from baseline | 76 | −3.3 ± 6.0 | 37 | −5.1 ± 6.0 | 39 | −1.5 ± 5.5 |
| Week 8 | 61 | 75.6 ± 10.8 | 32 | 75.8 ± 12.5 | 29 | 75.4 ± 8.7 |
| Change from baseline | 58 | −4.6 ± 6.2 | 30 | −6.4 ± 6.3 | 28 | −2.6 ± 5.5 |
| Week 12 | 67 | 76.6 ± 11.0 | 34 | 77.9 ± 12.9 | 33 | 75.4 ± 8.7 |
| Change from baseline | 65 | −4.2 ± 6.1*** | 32 | −5.7 ± 5.2*** | 33 | −2.8 ± 6.5* |
| EOT | 89 | 76.6 ± 10.3 | 44 | 76.9 ± 11.9 | 45 | 76.3 ± 8.5 |
| Change from baseline | 85 | −4.2 ± 5.9*** | 41 | −6.2 ± 5.3*** | 44 | −2.4 ± 5.9* |
| **Office SBP** | | | | | | |
| Baseline | 89 | 147.7 ± 18.0 | 44 | 146.3 ± 20.1 | 45 | 149.1 ± 15.6 |
| Week 4 | 84 | 134.8 ± 16.6 | 40 | 131.5 ± 17.3 | 44 | 137.7 ± 15.5 |
| Change from baseline | 84 | −12.5 ± 18.0 | 40 | −14.0 ± 17.9 | 44 | −11.2 ± 18.1 |
| Week 8 | 67 | 137.7 ± 15.7 | 34 | 133.3 ± 13.6 | 33 | 142.3 ± 16.6 |
| Change from baseline | 67 | −11.3 ± 17.0 | 34 | −12.8 ± 16.3 | 33 | −9.8 ± 17.7 |
| Week 12 | 79 | 136.4 ± 17.1 | 37 | 133.4 ± 17.6 | 42 | 139.0 ± 16.4 |
| Change from baseline | 79 | −11.0 ± 17.8*** | 37 | −12.0 ± 20.4** | 42 | −10.1 ± 15.2*** |
| EOT | 89 | 136.0 ± 17.3 | 44 | 133.2 ± 17.8 | 45 | 138.6 ± 16.5 |
| Change from baseline | 89 | −11.7 ± 17.9*** | 44 | −13.0 ± 20.4*** | 45 | −10.5 ± 15.3*** |
| **Office DBP** | | | | | | |
| Baseline | 89 | 84.4 ± 12.4 | 44 | 85.0 ± 12.9 | 45 | 83.7 ± 12.0 |
| Week 4 | 84 | 78.6 ± 12.7 | 40 | 78.7 ± 13.2 | 44 | 78.6 ± 12.4 |
| Change from baseline | 84 | −5.5 ± 9.2 | 40 | −6.4 ± 9.9 | 44 | −4.7 ± 8.6 |
| Week 8 | 67 | 79.3 ± 13.3 | 34 | 78.6 ± 11.4 | 33 | 80.2 ± 15.1 |
| Change from baseline | 67 | −5.5 ± 8.2 | 34 | −6.9 ± 8.3 | 33 | −4.0 ± 7.9 |
| Week 12 | 79 | 78.8 ± 12.4 | 37 | 79.5 ± 13.2 | 42 | 78.2 ±11.6 |
| Change from baseline | 79 | −5.1 ± 9.1*** | 37 | −5.4 ± 10.6** | 42 | −4.8 ± 7.8*** |
| EOT | 89 | 78.6 ± 12.7 | 44 | 78.8 ± 14.1 | 45 | 78.4 ± 11.4 |
| Change from baseline | 89 | −5.8 ± 9.9*** | 44 | −6.3 ± 11.6*** | 45 | −5.4 ± 8.0*** |

**p* < 0.05, ***p* < 0.01, ****p* < 0.001 vs. baseline, paired *t*-test.

*P*-values were not calculated for changes from baseline to Week 4 and Week 8.

*ARB* angiotensin receptor blocker, *BP* blood pressure, *CCB* calcium-channel blocker, *DBP* diastolic blood pressure, *EOT* end of treatment, *SBP* systolic blood pressure.

## Supplementary Table 4 Achievement rate of target BP^a^ levels at Week 12 (full analysis set)

|  | ***n*** | **%** | **95% CI** |
| --- | --- | --- | --- |
| Nighttime home BP (brachial) |  |  |  |
| Total (*N* = 93) | 16 | 17.2 | 10.2, 26.4 |
| ARB (*n* = 45) | 7 | 15.6 | 6.5, 29.5 |
| CCB (*n* = 48) | 9 | 18.8 | 8.9, 32.6 |
| Nighttime home BP (wrist) |  |  |  |
| Total (*N* = 93) | 28 | 30.1 | 21.0, 40.5 |
| ARB (*n* = 45) | 17 | 37.8 | 23.8, 53.5 |
| CCB (*n* = 48) | 11 | 22.9 | 12.0, 37.3 |
| Morning home BP |  |  |  |
| Total (*N* = 93) | 9 | 9.7 | 4.5, 17.6 |
| ARB (*n* = 45) | 4 | 8.9 | 2.5, 21.2 |
| CCB *(n* = 48) | 5 | 10.4 | 3.5, 22.7 |
| Bedtime home BP |  |  |  |
| Total (*N* = 93) | 24 | 25.8 | 17.3, 35.9 |
| ARB (*n* = 45) | 14 | 31.1 | 18.2, 46.6 |
| CCB (*n* = 48) | 10 | 20.8 | 10.5, 35.0 |
| Office BP |  |  |  |
| Total (*N* = 93) | 24 | 25.8 | 17.3, 35.9 |
| ARB (*n* = 45) | 13 | 28.9 | 16.4, 44.3 |
| CCB (*n* = 48) | 11 | 22.9 | 12.0, 37.3 |

^a^The achievement rate of target BP levels was defined as office BP (<130/80 mmHg), home BP (<125/75 mmHg), and nighttime home BP (<120/70 mmHg) at 12 weeks.

95% CIs were calculated using the Clopper–Pearson method.

*ARB* angiotensin receptor blocker, *BP* blood pressure, *CCB* calcium-channel blocker, *CI* confidence interval.

## Supplementary Table 5 Achievement rate of target BP^a^ levels at Week 12 (per-protocol set)

|  | ***n*** | **%** | **95% CI** |
| --- | --- | --- | --- |
| Nighttime home BP (brachial) |  |  |  |
| Total (*N* = 89) | 16 | 18.0 | 10.6, 27.5 |
| ARB (*n* = 44) | 7 | 15.9 | 6.6, 30.1 |
| CCB (*n* = 45) | 9 | 20.0 | 9.6, 34.6 |
| Nighttime home BP (wrist) |  |  |  |
| Total (*N* = 89) | 27 | 30.3 | 21.0, 41.0 |
| ARB (*n* = 44) | 16 | 36.4 | 22.4, 52.2 |
| CCB (*n* = 45) | 11 | 24.4 | 12.9, 39.5 |
| Morning home BP |  |  |  |
| Total (*N* = 89) | 8 | 9.0 | 4.0, 16.9 |
| ARB (*n* = 44) | 4 | 9.1 | 2.5, 21.7 |
| CCB (*n* = 45) | 4 | 8.9 | 2.5, 21.2 |
| Bedtime home BP |  |  |  |
| Total (*N* = 89) | 23 | 25.8 | 17.1, 36.2 |
| ARB (*n* = 44) | 13 | 29.5 | 16.8, 45.2 |
| CCB (*n* = 45) | 10 | 22.2 | 11.2, 37.1 |
| Office BP |  |  |  |
| Total (*N* = 89) | 23 | 25.8 | 17.1, 36.2 |
| ARB (*n* = 44) | 12 | 27.3 | 15.0, 42.8 |
| CCB (*n* = 45) | 11 | 24.4 | 12.9, 39.5 |

^a^The achievement rate of target BP levels was defined as office BP (<130/80 mmHg), home BP (<125/75 mmHg), and nighttime home BP (<120/70 mmHg) at 12 weeks.

95% CIs were calculated using the Clopper–Pearson method.

*ARB* angiotensin receptor blocker, *BP* blood pressure, *CCB* calcium-channel blocker, *CI* confidence interval.

## Supplementary Table 6 Change in biomarker data from baseline to Week 12 in the total population and ARB and CCB subcohorts (full analysis set)

|  |  | **Total**  ***N* = 93** | **ARB subcohort**  ***n* = 45** | **CCB subcohort**  ***n* = 48** |
| --- | --- | --- | --- | --- |
| **UACR, mg/gCr** |  |  |  |  |
| Baseline | Mean ± SD | *N* = 93  110.4 ± 321.5 | *n* = 45  121.4 ± 302.5 | *n* = 48  100.1 ± 341.1 |
| Week 12 | Mean ± SD | *N* = 81  72.0 ± 324.7 | *n* = 38  117.6 ± 469.4 | *n* = 43  31.8 ± 56.4 |
|  | Change from baseline | *N* = 81  −38.7 ± 271.7 | *n* = 38  −3.7 ± 206.8 | *n* = 43  −69.6 ± 317.6 |
|  | Geometric percentage change from baseline  95% CI | *N* = 81  −26.2***  −37.0, −13.7 | *n* = 38  −29.9**  −44.4, −11.6 | *n* = 43  −22.8*  −38.2, −3.6 |
| **NT-proBNP, pg/ml** |  |  |  |  |
| Baseline | Mean ± SD | *N* = 93  83.7 ± 92.3 | *n* = 45  79.6 ± 75.3 | *n* = 48  87.4 ± 106.5 |
| Week 12 | Mean ± SD | *N* = 81  87.0 ± 161.0 | *n* = 38  92.4 ± 200.8 | *n* = 43  82.2 ± 117.5 |
|  | Change from baseline | *N* = 81  1.3 ± 137.9 | *n* = 38  12.8 ± 196.9 | *n* = 43  −8.9 ± 43.0 |
|  | Geometric percentage change from baseline  95% CI | *N* = 81  −18.5**  −28.5, −7.1 | *n* = 38  −18.3  −35.7, 3.7 | *n* = 43  −18.6**  −29.1, −6.6 |
| **PAC, pg/ml** |  |  |  |  |
| Baseline | Mean ± SD | *N* = 93  39.4 ± 36.8 | *n* = 45  28.5 ± 23.2 | *n* = 48  49.7 ± 43.8 |
| Week 12 | Mean ± SD | *N* = 81  84.9 ± 68.6 | *n* = 38  57.4 ± 45.5 | *n* = 43  109.2 ± 76.5 |
|  | Change from baseline | *N* = 81  47.3 ± 55.3 | *n* = 38  26.1 ± 35.5 | *n* = 43  66.0 ± 62.8 |
| **PRA, ng/ml/hr** |  |  |  |  |
| Baseline | Mean ± SD | *N* = 93  2.7 ± 5.8 | *n* = 45  4.2 ± 8.0 | *n* = 48  1.3 ± 1.4 |
| Week 12 | Mean ± SD | *N* = 81  6.6 ± 10.8 | *n* = 38  10.5 ± 14.6 | *n* = 43  3.1 ± 3.1 |
|  | Change from baseline | *N* = 81  3.8 ± 9.4 | *n* = 38  5.8 ± 13.3 | *n* = 43  1.9 ± 2.3 |
| **Urinary sodium, mEq/l** |  |  |  |  |
| Baseline | Mean ± SD | *N* = 93  113.5 ± 56.1 | *n* = 45  107.6 ± 55.3 | *n* = 48  119.0 ± 56.9 |
| Week 12 | Mean ± SD | *N* = 81  116.0 ± 53.6 | *n* = 38  113.4 ± 51.7 | *n* = 43  118.2 ± 55.8 |
|  | Change from baseline | *N* = 81  3.8 ± 50.9 | *n* = 38  10.2 ± 50.2 | *n* = 43  −1.8 ± 51.4 |
| **Urinary potassium, mEq/l** |  |  |  |  |
| Baseline | Mean ± SD | *N* = 93  43.9 ± 29.7 | *n* = 45  50.5 ± 35.3 | *n* = 48  37.7 ± 21.9 |
| Week 12 | Mean ± SD | *N* = 81  44.7 ± 24.8 | *n* = 38  44.2 ± 22.4 | *n* = 43  45.1 ± 27.1 |
|  | Change from baseline | *N* = 81  0.1 ± 28.9 | *n* = 38  −7.9 ± 30.4 | *n* = 43  7.1 ± 25.7 |
| **Urinary sodium/potassium** |  |  |  |  |
| Baseline | Mean ± SD | *N* = 93  3.6 ± 3.3 | *n* = 45  2.9 ± 1.8 | *n* = 48  4.3 ± 4.1 |
| Week 12 | Mean ± SD | *N* = 81  3.4 ± 2.5 | *n* = 38  3.2 ± 2.1 | *n* = 43  3.5 ± 2.8 |
|  | Change from baseline | *N* = 81  −0.3 ± 3.1 | *n* = 38  0.4 ± 1.9 | *n* = 43  −0.8 ± 3.9 |

**p* < 0.05, ***p* < 0.01, ****p* < 0.001 vs. baseline, paired *t*-test.

*P*-values were calculated only for geometric percentage change from baseline to week 12, and not for change from baseline to Week 12.

*ARB* angiotensin receptor blocker, *CCB* calcium-channel blocker, *CI* confidence interval, *NT-proBNP* N-terminal pro-brain natriuretic peptide, *PAC* plasma aldosterone concentration, *PRA* plasma renin activity, *UACR* urinary albumin-to-creatinine ratio.

## Supplementary Table 7 Change in biomarker data from baseline to Week 12 in the total population and ARB and CCB subcohorts (per-protocol set)

|  |  | **Total**  ***N* = 89** | **ARB subcohort**  ***n* = 44** | **CCB subcohort**  ***n* = 45** |
| --- | --- | --- | --- | --- |
| **UACR, mg/gCr** |  |  |  |  |
| Baseline | Mean ± SD | *N* = 89  109.8 ± 327.9 | *n* = 44  122.2 ± 306.0 | *n* = 45  97.6 ± 351.0 |
| Week 12 | Mean ± SD | *N* = 78  72.8 ± 330.8 | *n* = 37  118.0 ± 475.8 | *n* = 41  32.0 ± 57.8 |
|  | Change from baseline | *N* = 78  −39.8 ± 276.8 | *n* = 37  −4.3 ± 209.6 | *n* = 41  −71.7 ± 325.2 |
|  | Geometric percentage change from baseline  95% CI | *N* = 78  −26.1***  −37.1, −13.2 | *n* = 37  −30.9**  −45.5, −12.5 | *n* = 41  −21.5*  −37.5, −1.4 |
| **NT-proBNP, pg/ml** |  |  |  |  |
| Baseline | Mean ± SD | *N* = 89  84.2 ± 93.9 | *n* = 44  80.5 ± 76.0 | *n* = 45  87.9 ± 109.4 |
| Week 12 | Mean ± SD | *N* = 78  88.7 ± 163.8 | *n* = 37  94.3 ± 203.2 | *n* = 41  83.6 ± 120.0 |
|  | Change from baseline | *N* = 78  1.6 ± 140.5 | *n* = 37  13.7 ± 199.5 | *n* = 41  −9.3 ± 44.0 |
|  | Geometric percentage change from baseline  95% CI | *N* = 78  −18.5**  −28.8, −6.6 | *n* = 37  −17.4  −35.3, 5.5 | *n* = 41  −19.4**  −30.2, −6.9 |
| **PAC, pg/ml** |  |  |  |  |
| Baseline | Mean ± SD | *N* = 89  40.1 ± 36.8 | *n* = 44  29.0 ± 23.3 | *n* = 45  50.9 ± 43.9 |
| Week 12 | Mean ± SD | *N* = 78  85.8 ± 68.7 | *n* = 37  58.1 ± 45.9 | *n* = 41  110.9 ± 76.5 |
|  | Change from baseline | *N* = 78  48.0 ± 56.0 | *n* = 37  26.2 ± 36.0 | *n* = 41  67.7 ± 63.5 |
| **PRA, ng/ml/hr** |  |  |  |  |
| Baseline | Mean ± SD | *N* = 89  2.7 ± 5.8 | *n* = 44  4.0 ± 8.0 | *n* = 45  1.4 ± 1.4 |
| Week 12 | Mean ± SD | *N* = 78  6.5 ± 10.8 | *n* = 37  10.2 ± 14.7 | *n* = 41  3.3 ± 3.1 |
|  | Change from baseline | *N* = 78  3.8 ± 9.6 | *n* = 37  5.7 ± 13.5 | *n* = 41  2.0 ± 2.3 |
| **Urinary sodium, mEq/l** |  |  |  |  |
| Baseline | Mean ± SD | *N* = 89  113.3 ± 56.8 | *n* = 44  106.5 ± 55.5 | *n* = 45  120.1 ± 58.0 |
| Week 12 | Mean ± SD | *N* = 78  115.3 ± 53.8 | *n* = 37  111.6 ± 51.1 | *n* = 41  118.6 ± 56.5 |
|  | Change from baseline | *N* = 78  3.4 ± 51.8 | *n* = 37  9.8 ± 50.9 | *n* = 41  −2.4 ± 52.6 |
| **Urinary potassium, mEq/l** |  |  |  |  |
| Baseline | Mean ± SD | *N* = 89  44.0 ± 29.9 | *n* = 44  49.9 ± 35.5 | *n* = 45  38.3 ± 22.2 |
| Week 12 | Mean ± SD | *N* = 78  44.0 ± 24.7 | *n* = 37  42.9 ± 21.2 | *n* = 41  45.0 ± 27.8 |
|  | Change from baseline | *N* = 78  −0.5 ± 29.1 | *n* = 37  −8.5 ± 30.6 | *n* = 41  6.7 ± 26.0 |
| **Urinary sodium/potassium** |  |  |  |  |
| Baseline | Mean ± SD | *N* = 89  3.6 ± 3.3 | *n* = 44  2.9 ± 1.9 | *n* = 45  4.3 ± 4.2 |
| Week 12 | Mean ± SD | *N* = 78  3.4 ± 2.5 | *n* = 37  3.2 ± 2.1 | *n* = 41  3.6 ± 2.8 |
|  | Change from baseline | *N* = 78  −0.2 ± 3.2 | *n* = 37  0.4 ± 2.0 | *n* = 41  −0.8 ± 3.9 |

**p* < 0.05, ***p* < 0.01, ****p* < 0.001 vs. baseline, paired *t*-test.

*P*-values were calculated only for geometric percentage change from baseline to week 12, and not for change from baseline to Week 12.

*ARB* angiotensin receptor blocker, *CCB* calcium-channel blocker, *CI* confidence interval, *NT-proBNP* N-terminal pro-brain natriuretic peptide, *PAC* plasma aldosterone concentration, *PRA* plasma renin activity, *UACR* urinary albumin-to-creatinine ratio.

## Supplementary Table 8 Change in CAVI from baseline to Week 12 (full analysis set)

|  |  | **Total**  ***N* = 93** | **ARB subcohort**  ***n* = 45** | **CCB subcohort**  ***n* = 48** |
| --- | --- | --- | --- | --- |
| **CAVI** |  |  |  |  |
| Baseline | Mean ± SD | *N* = 54  9.3 ± 1.4 | *n* = 29  9.5 ± 1.3 | *n* = 25  9.1 ± 1.5 |
| Week 12 | Mean ± SD | *N* = 48  9.1 ± 1.5 | *n* = 24  9.2 ± 1.4 | *n* = 24  9.0 ± 1.6 |
|  | Change from baseline | *N* = 48  −0.2 ± 0.7 | *n* = 24  −0.3 ± 0.7 | *n* = 24  −0.1 ± 0.7 |
|  | Geometric percentage change from baseline  95% CI | *N* = 48  −2.2*  −4.3, −0.1 | *n* = 24  −3.3*  −6.3, −0.3 | *n* = 24  −1.1  −4.3, 2.1 |

**p* < 0.05 vs. baseline, paired *t*-test.

*P*-values were not calculated for change from baseline to Week 12.

*ARB* angiotensin receptor blocker, *CAVI* cardio-ankle vascular index, *CCB* calcium-channel blocker, *CI* confidence interval.

## Supplementary Table 9 Change in CAVI from baseline to Week 12 (per-protocol set)

|  |  | **Total**  ***N* = 89** | **ARB subcohort**  ***n* = 44** | **CCB subcohort**  ***n* = 45** |
| --- | --- | --- | --- | --- |
| **CAVI** |  |  |  |  |
| Baseline | Mean ± SD | *N* = 50  9.3 ± 1.4 | *n* = 28  9.6 ± 1.3 | *n* = 22  9.0 ± 1.5 |
| Week 12 | Mean ± SD | *N* = 45  9.0 ± 1.5 | *n* = 23  9.2 ± 1.5 | *n* = 22  8.8 ± 1.6 |
|  | Change from baseline | *N* = 45  −0.2 ± 0.7 | *n* = 23  −0.3 ± 0.7 | *n* = 22  −0.2 ± 0.7 |
|  | Geometric percentage change from baseline  95% CI | *N* = 45  −2.7*  −4.9, −0.5 | *n* = 23  −3.5*  −6.6, −0.4 | *n* = 22  −1.9  −5.1, 1.4 |

**p* < 0.05 vs. baseline, paired *t*-test.

*P*-values were not calculated for change from baseline to Week 12.

*ARB* angiotensin receptor blocker, *CAVI* cardio-ankle vascular index, *CCB* calcium-channel blocker, *CI* confidence interval.

## Supplementary **Table 10** Incidence of serum potassium level ≥5.5 and ≥6.0 mEq/l (safety analysis set)

|  | **Total**  ***N* = 101** | **ARB subcohort**  ***n* = 48** | **CCB subcohort**  ***n* = 53** |
| --- | --- | --- | --- |
| Serum potassium ≥5.5 mEq/l | 11 (10.9) | 9 (18.8) | 2 (3.8) |
| Serum potassium ≥6.0 mEq/l | 2 (2.0) | 2 (4.2) | 0 |

Data are n (%).

*ARB* angiotensin receptor blocker, *CCB* calcium-channel blocker.
