# Supplementary material for: Nighttime home blood pressure lowering effect of esaxerenone in patients with uncontrolled nocturnal hypertension: the EARLY-NH study
Source: Hypertens Res. 2023 May 12;46(7):1782–94. doi: 10.1038/s41440-023-01292-0 (PMC10319630; doi:10.1038/s41440-023-01292-0)
Supplement: Supplementary file 2 — Supplementary Figures [file 41440_2023_1292_MOESM2_ESM.pptx]

## Slide 1
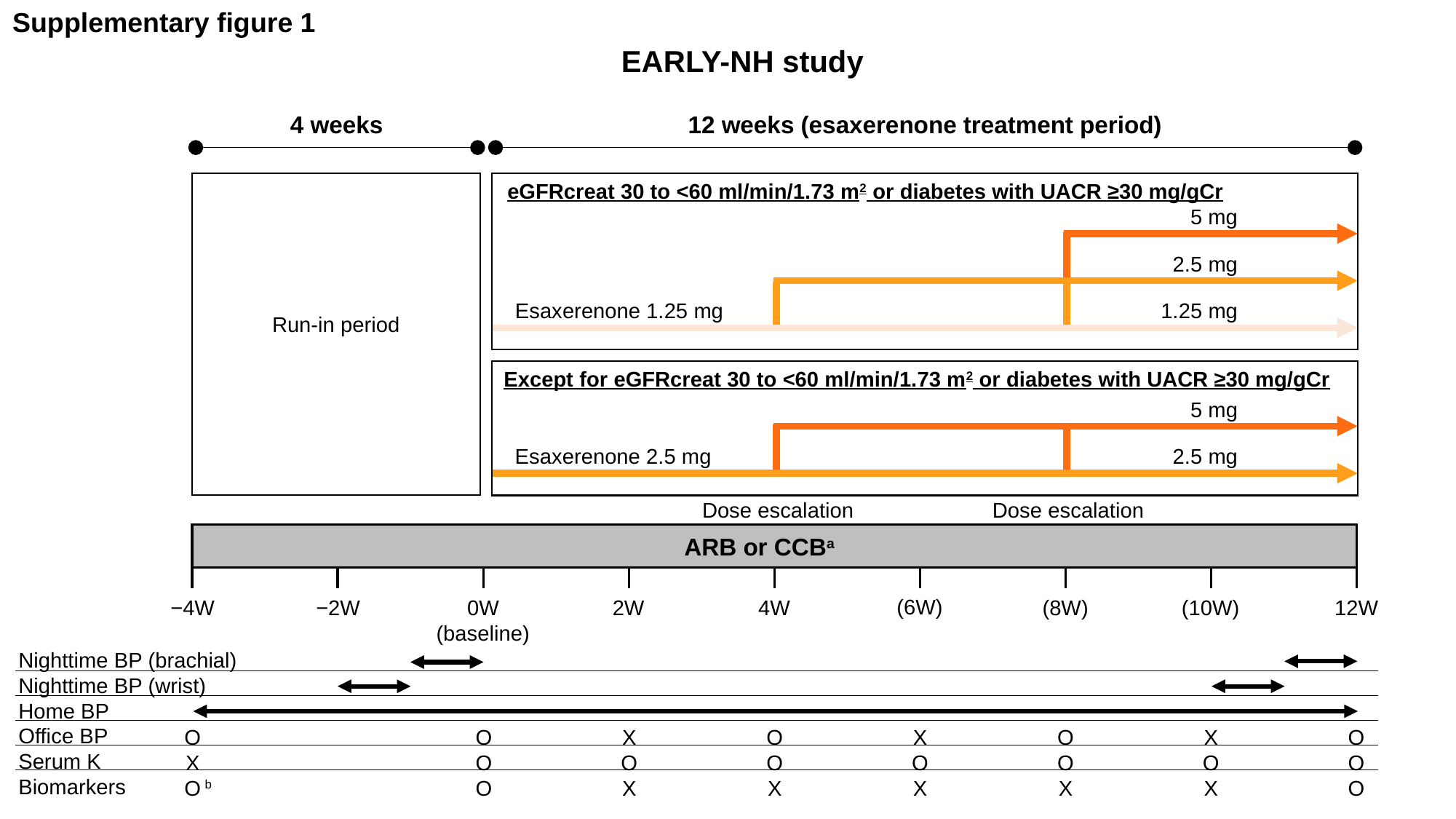

Supplementary figure 1
EARLY-NH study
4 weeks
12 weeks (esaxerenone treatment period)
Run-in period
eGFRcreat 30 to <60 ml/min/1.73 m2 or diabetes with UACR ≥30 mg/gCr
5 mg
2.5 mg
1.25 mg
Esaxerenone 1.25 mg
Except for eGFRcreat 30 to <60 ml/min/1.73 m2 or diabetes with UACR ≥30 mg/gCr
5 mg
Esaxerenone 2.5 mg
2.5 mg
Dose escalation
Dose escalation
ARB or CCBa
−4W
−2W
0W
(baseline)
2W
4W
(6W)
(8W)
(10W)
12W
| |
| --- |
| |
| |
| |
| |
| |
Nighttime BP (brachial)
Nighttime BP (wrist)
Home BP
Office BP
Serum K
Biomarkers
O
X
O
O
O
O
X
O
X
O
O
X
X
O
X
O
O
X
X
O
X
O
O
O
b

## Slide 2
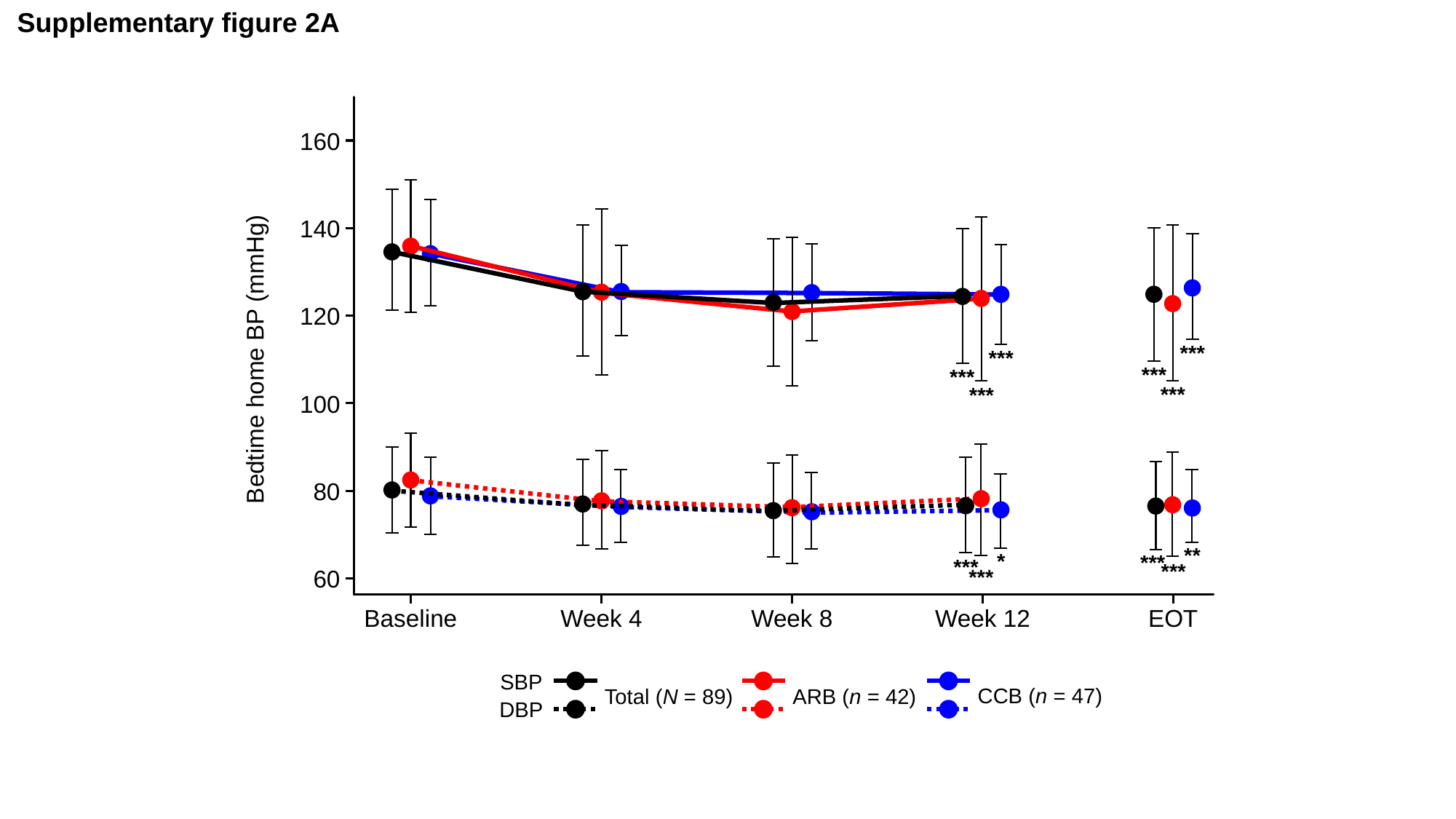

Supplementary figure 2A
160
***
***
***
***
***
***
140
120
Bedtime home BP (mmHg)
100
***
***
**
*
80
60
Baseline
Week 4
Week 8
Week 12
EOT
SBP
DBP
Total (N = 89)
ARB (n = 42)
CCB (n = 47)
***
***

## Slide 3
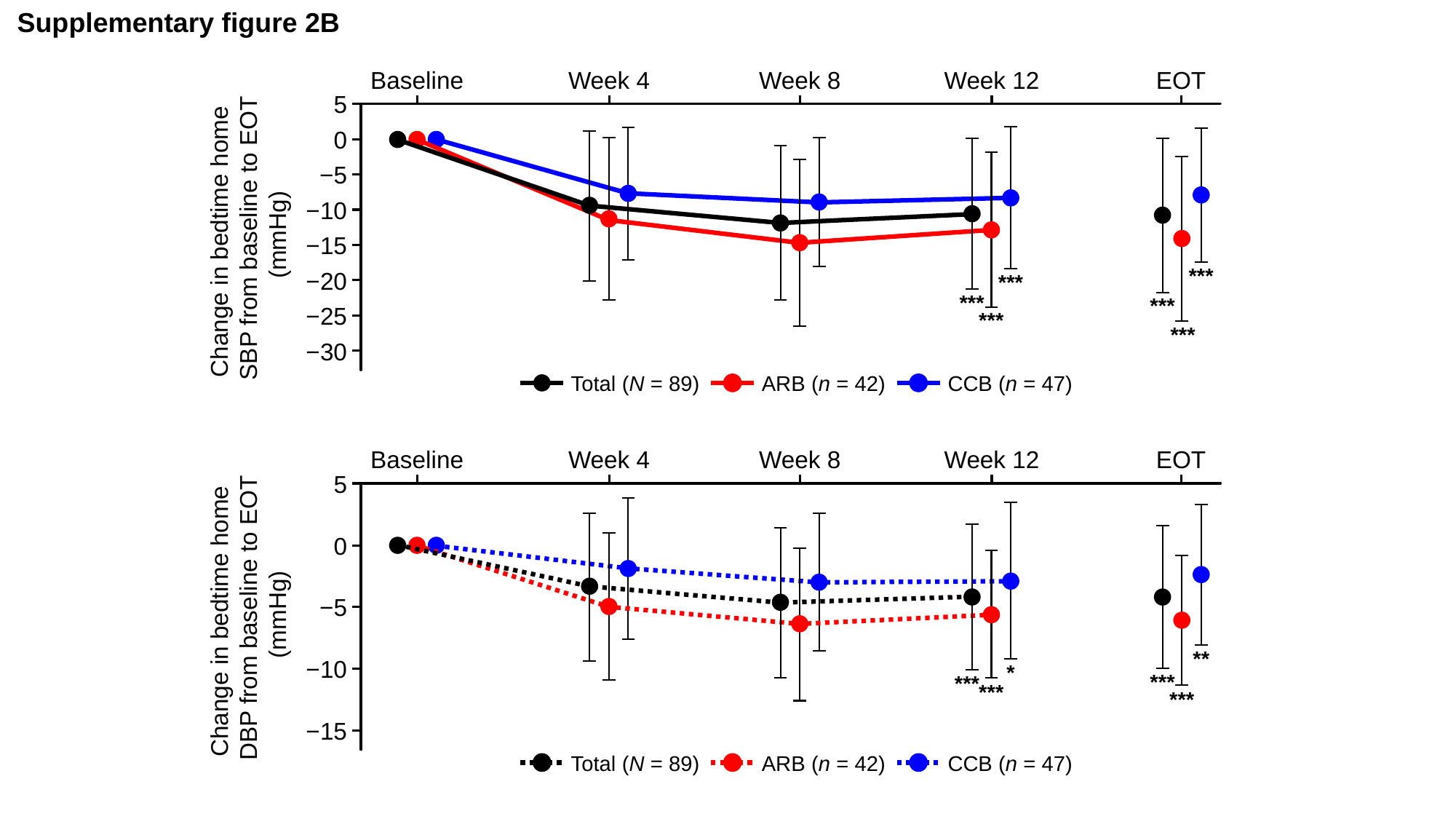

Supplementary figure 2B
Baseline
Week 4
Week 8
Week 12
EOT
5
0
***
***
***
***
***
−5
Change in bedtime home
SBP from baseline to EOT
 (mmHg)
−10
−15
***
−20
−25
−30
Total (N = 89)
ARB (n = 42)
CCB (n = 47)
Baseline
Week 4
Week 8
Week 12
EOT
5
*
**
***
0
***
***
Change in bedtime home
DBP from baseline to EOT
 (mmHg)
−5
−10
***
−15
Total (N = 89)
ARB (n = 42)
CCB (n = 47)

## Slide 4
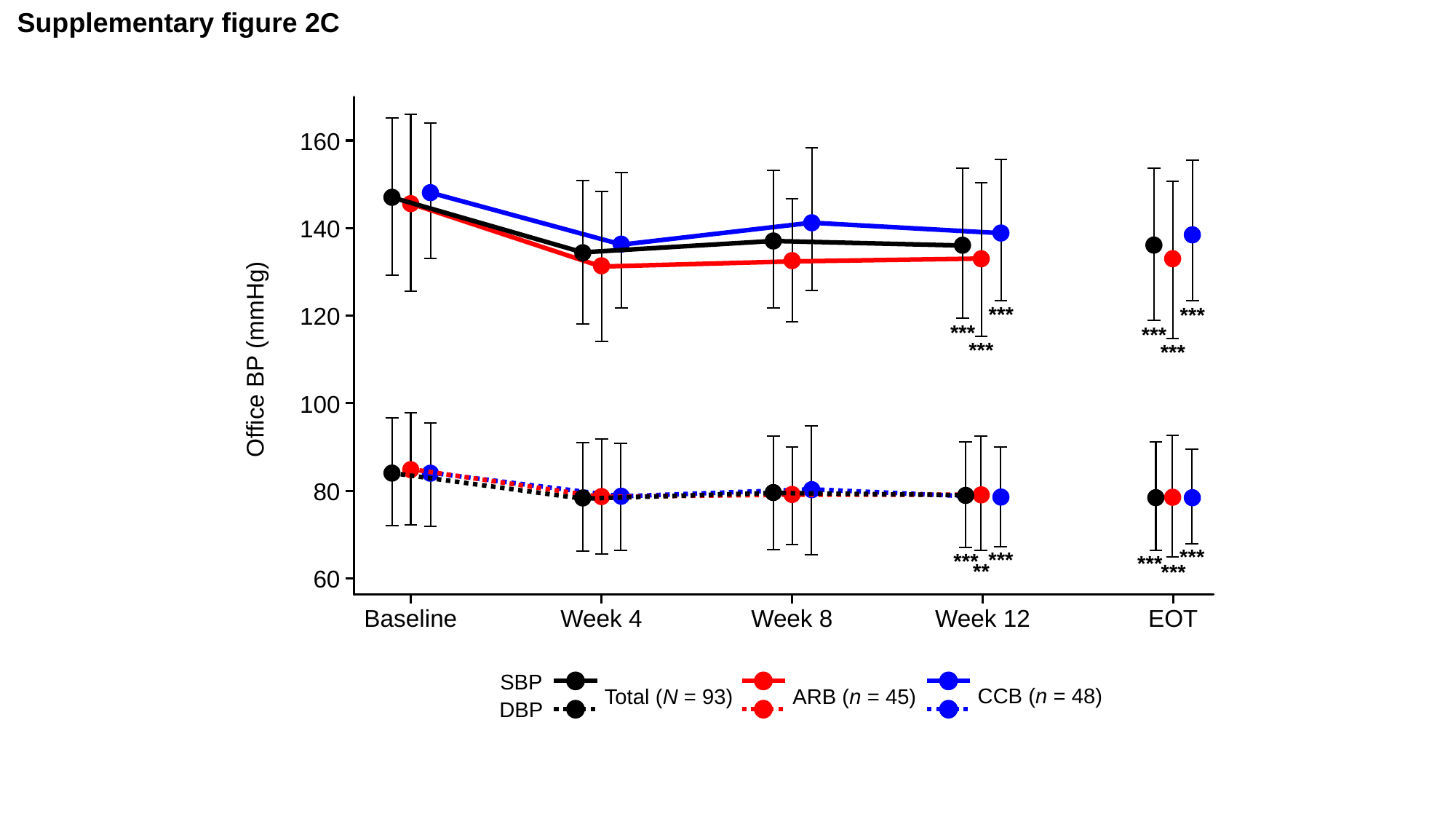

Supplementary figure 2C
***
***
***
***
***
***
160
140
120
Office BP (mmHg)
100
***
**
***
***
***
***
80
60
Baseline
Week 4
Week 8
Week 12
EOT
SBP
DBP
Total (N = 93)
ARB (n = 45)
CCB (n = 48)

## Slide 5
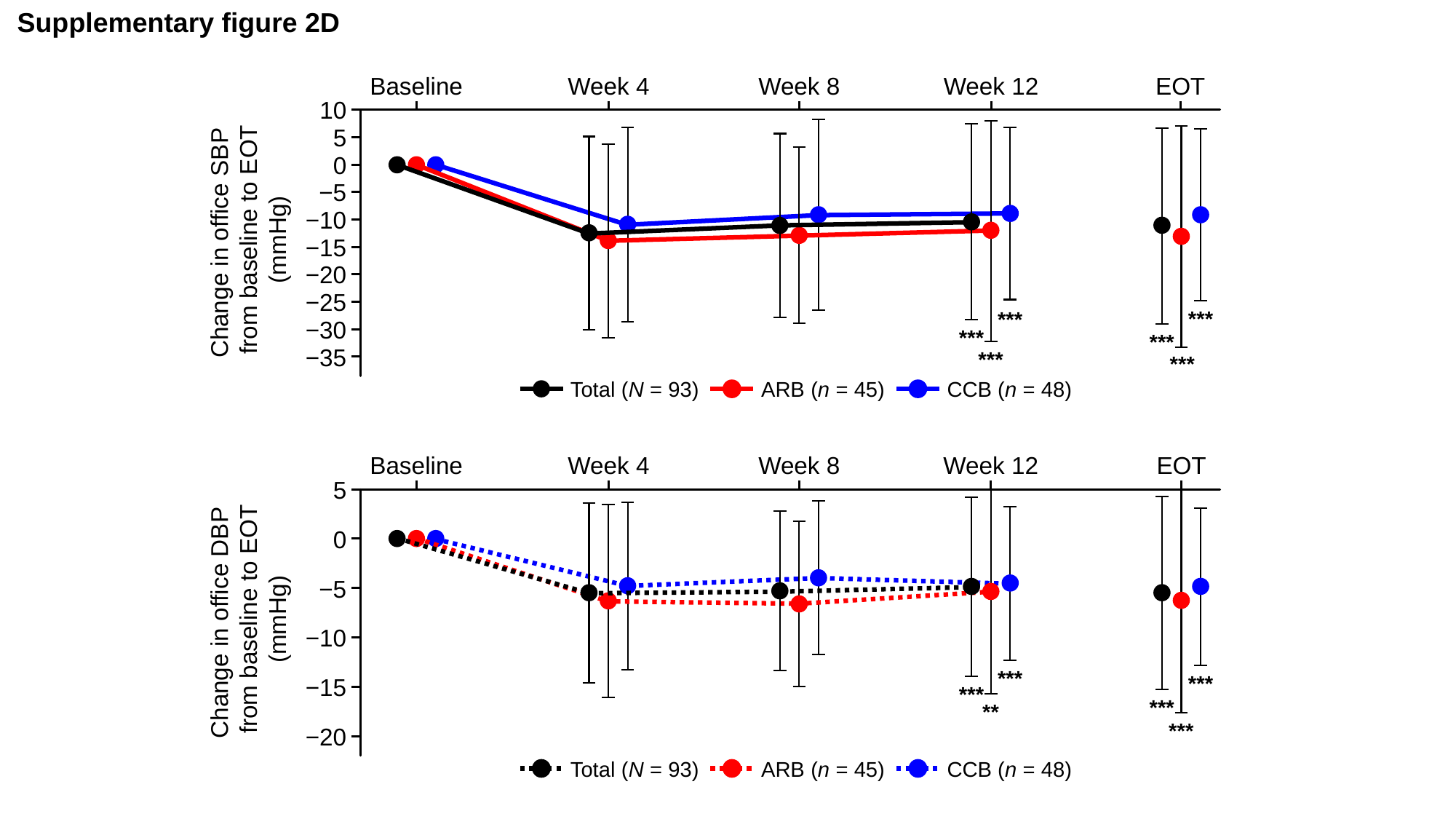

Supplementary figure 2D
Baseline
Week 4
Week 8
Week 12
EOT
10
***
***
***
***
5
***
***
0
−5
Change in office SBP
 from baseline to EOT
 (mmHg)
−10
−15
−20
−25
−30
−35
Total (N = 93)
ARB (n = 45)
CCB (n = 48)
Baseline
Week 4
Week 8
Week 12
EOT
5
**
***
***
***
***
***
0
Change in office DBP
 from baseline to EOT
 (mmHg)
−5
−10
−15
−20
Total (N = 93)
ARB (n = 45)
CCB (n = 48)
