# Supplementary material for: Nighttime home blood pressure lowering effect of esaxerenone in patients with uncontrolled nocturnal hypertension: the EARLY-NH study
Source: Hypertens Res. 2023 May 12;46(7):1782–94. doi: 10.1038/s41440-023-01292-0 (PMC10319630; doi:10.1038/s41440-023-01292-0)
Supplement: Supplementary file 3 — Supplementary Figure legends [file 41440_2023_1292_MOESM3_ESM.docx]

# Supplementary figure legends

**Supplementary Figure 1** Study design.

**^a^**The following concomitant drugs were prohibited from 4 weeks before the start of the run-in period to the end of treatment or discontinuation: antihypertensive drugs (ARBs [if taking a CCB], angiotensin converting enzyme inhibitors, CCBs [if taking an ARB], α-blockers, β-blockers, other sympatholytic agents, vasodilators, and renin inhibitors); diuretics (thiazide diuretics, thiazide-like diuretics, loop diuretics, potassium sparing diuretics); aldosterone antagonists; angiotensin receptor neprilysin inhibitors; potassium preparations; serum potassium inhibitors and hyperkalemia-improving agents; and herbal medicine containing liquorice.

**^b^**Only urinary markers were measured at Week −4 (both serum and urinary markers were measured at Weeks 0 and 12).

ARB angiotensin receptor blocker, BP blood pressure, CCB calcium-channel blocker, eGFRcreat creatinine-based estimated glomerular filtration rate, K potassium, UACR urinary albumin-to-creatinine ratio, W weeks

**Supplementary Figure 2** Time course changes and changes from baseline in bedtime home BP (**A, B**) and office BP (**C, D**) throughout the study period in the total population and ARB or CCB subcohorts (full analysis set).

Data are mean ± SD.

**p* < 0.05, ***p* < 0.01, ****p* < 0.001 vs. baseline, paired *t*-test.

ARB angiotensin receptor blocker, BP blood pressure, CCB calcium-channel blocker, DBP diastolic blood pressure, EOT end of treatment, SBP systolic blood pressure
